# Emerging prospects of mRNA cancer vaccines: mechanisms, formulations, and challenges in cancer immunotherapy

**DOI:** 10.3389/fimmu.2024.1448489

**Published:** 2024-11-25

**Authors:** Umm E. Laila, Wang An, Zhi-Xiang Xu

**Affiliations:** School of Life Sciences, Henan University, Kaifeng, Henan, China

**Keywords:** mRNA, vaccine, immunotherapy, cancer, immune response

## Abstract

Cancer continues to pose an alarming threat to global health, necessitating the need for the development of efficient therapeutic solutions despite massive advances in the treatment. mRNA cancer vaccines have emerged as a hopeful avenue, propelled by the victory of mRNA technology in COVID-19 vaccines. The article delves into the intricate mechanisms and formulations of cancer vaccines, highlighting the ongoing efforts to strengthen mRNA stability and ensure successful translation inside target cells. Moreover, it discusses the design and mechanism of action of mRNA, showcasing its potential as a useful benchmark for developing efficacious cancer vaccines. The significance of mRNA therapy and selecting appropriate tumor antigens for the personalized development of mRNA vaccines are emphasized, providing insights into the immune mechanism. Additionally, the review explores the integration of mRNA vaccines with other immunotherapies and the utilization of progressive delivery platforms, such as lipid nanoparticles, to improve immune responses and address challenges related to immune evasion and tumor heterogeneity. While underscoring the advantages of mRNA vaccines, the review also addresses the challenges associated with the susceptibility of RNA to degradation and the difficulty in identifying optimum tumor-specific antigens, along with the potential solutions. Furthermore, it provides a comprehensive overview of the ongoing research efforts aimed at addressing these hurdles and enhancing the effectiveness of mRNA-based cancer vaccines. Overall, this review is a focused and inclusive impression of the present state of mRNA cancer vaccines, outlining their possibilities, challenges, and future predictions in the fight against cancer, ultimately aiding in the development of more targeted therapies against cancer.

## 1 Introduction

Cancer remains a significant global health challenge, with estimates projecting 34 million new cases by 2070 (1). Despite advancements in treatment, current therapies exhibit limitations, emphasizing the need for innovative solutions for cancer treatment. (2). The concept of mRNA cancer vaccines has emerged as a promising avenue for immunotherapy, following the success of mRNA technology in producing effective COVID-19 vaccines during the pandemic. (3–6). Immunotherapy, such as immune checkpoint inhibitors and CAR-T therapies, have revolutionized cancer treatment by harnessing the power of the immune system. (7). Recently, these approaches, combined with mRNA vaccines, offer a personalized and effective strategy against cancer, providing a more targeted approach (8, 9).

The mRNA vaccines offer a novel prophylactic strategy, delivering genetic instructions directly to the cells for inducing precise protein production and triggering robust immune responses to actively combat cancer (10). A typical mRNA vaccine harbors synthetic mRNA molecules designed to encode cancer-specific antigens (11). In this regard, various optimization strategies are employed to ensure efficient translation of mRNA vaccines within human cells (12). The advent of lipid nanoparticles, frequently employed as delivery vehicles to encapsulate mRNA to prevent it from degradation and facilitate cellular uptake, has significantly revolutionized the field of mRNA vaccines (13). Once inside the cells, the mRNA gets translated into the antigen protein by utilizing the cellular translation machinery (14). Consequently, the antigen protein gets processed by the antigen-presenting cells and presented to the immune cells for eliciting a robust innate and adaptive immune response (15). The successful development of the first mRNA cancer vaccine in 1995, which encoded the carcinoembryonic antigen in mice, marked a significant milestone, which prompted scientists to critically explore the potential of immunotherapy against cancer (16). The mRNA vaccines hold a distinctive advantage over conventional virus-based vaccines, owing to their enhanced safety, cost-effectiveness, purity, dismal vaccine resistance, and integration concerns (17). Moreover, the mRNA vaccines encoding full-length tumor antigens can generate broad-spectrum T-cell-mediated immune responses regardless of the Human Leucocyte Antigen (HLA) types (18, 19), hence elevating their therapeutic potential (20). Furthermore, multiple strategies, including mRNA encapsulation in the immune cell-specific nanoparticles and self-amplifying mRNA (saRNA) designs, aim to improve vaccine efficacy and minimize the potential side effects (21, 22). Additionally, novel approaches involving the incorporation of mRNAs encoding multiple antigens in the vaccine are substantially explored to broaden the protection against various diseases (23). The remarkable advancements in mRNA vaccine technology hold great promise for addressing health concerns through the practical utility of these vaccines in nearby future.

The current review aims to emphasize the significance of mRNA vaccines as an innovative immunotherapeutic approach against cancer. It will address the intricate mechanisms and formulations of these groundbreaking vaccines, highlighting their effectiveness, mechanism of immune system activation, and future safety considerations. As the field of mRNA vaccines continues to expand, the upcoming research strives to optimize these vaccines by improving the efficacy, minimizing the side effects, addressing the inherent susceptibility of mRNA to enzymatic degradation, and ensuring successful mRNA translation in the target cells. Consequently, these attempts will remarkably elevate the applicability of mRNA vaccines against diverse cancers.

## 2 mRNA vaccine as a therapeutic tool for cancer immunotherapy

Recently, mRNA, a molecule central to cellular protein translation, has emerged as a notable platform in remarkably revolutionizing cancer immunotherapy. Compared to DNA-based vaccines, mRNA vaccines offer marked benefits making them a promising therapeutic choice (24). In this regard, mRNA vaccines harbor a potentially enhanced therapeutic effectiveness due to their equivalent tendency of translation in the dividing and non-dividing cells. Moreover, unlike DNA-based vaccines, mRNA vaccines evade the requirement of integration into the host genome, and showcase their effects via cytosolic translation of Tumor-Associated Antigens (TAA), hence minimizing the subsequent cellular damage. Consequently, mRNA appears to be an exceptionally competent candidate for vaccine development. Despite abundant advantages, the challenges regarding the mode of delivery, stability, and specificity of mRNA vaccines persist. Therefore, comprehensive research is crucial to address these challenges and fully harness the potential of this impressive technology for targeting cancer. **Table 1** illustrates an extensive review of the historical aspects encompassing the advent and advancement of mRNA vaccine technology for cancer.

**Table 1:** Timeline of the advent and advancements of the mRNA vaccine technology adapted by Li et al. (25).

Year	Milestone
1961	Discovery of messenger RNA (mRNA)
1963	The discovery that interferon is inducible by mRNA
1965	First liposome produced
1969	First-time protein isolation from mRNA in the lab
1974	Liposomes utilized for vaccine delivery
1975	Identification of the 5’ Cap modification in mRNA
1978	First delivery of mRNA wrapped in liposomes to cells
1984	Synthesis of mRNA in a laboratory setting
1989-1990	Proposal of mRNA vaccine concept; synthetic mRNA delivered to human cells and frog embryos
1992	Discovery of cancer therapy through inhibition of negative immune regulation
1993	mRNA found to induce both cellular and humoral immunity
1994	Introduction of self-replicating mRNA
1995	mRNA tested as a cancer vaccine in mice
2004	Identification of protamine-stabilized RNA as a potent danger signal
2005	The discovery that modified RNA can evade immune detection
2008	Phase I/II trials with mRNA vaccine Ractive in melanoma and non-small cell lung cancer (NSCLC) patients
2009	First direct injection of mRNA in human cancer
2010	Intranodal delivery of mRNA transfects DCs and elicits antitumor immunity
2012	First mRNA vaccines in lipid nanoparticles tested in mice
2013	Debate on type I INFs in efficacy & safety of mRNA vaccines
2015	Phase I/II trials with mRNA vaccine Ractive in melanoma and non-small cell lung cancer (NSCLC) patients
2016	Anti-tumor immunity by intravenous delivery of mRNA LNPs with type I IFN as a driving force
2017	Initial human trials of conceptual personalized mRNA cancer vaccines
2018	The first drug with lipid nanoparticles (patisiran) approved
2019	Clinical trials with RNA vaccines for infections & cancer, trends toward LNP-based delivery; combination with checkpoints
2021	Impact of SARS-CoV-2 mRNA vaccines on tumor patients and potential anti-tumor effects
2022	Increased focus on personalized mRNA cancer vaccines targeting neoantigens
2023	Promising results from Phase II and III trials of mRNA cancer vaccines
2024	Ongoing Phase III trials and potential for first regulatory approvals of personalized mRNA cancer vaccines

### 2.1 Construction of synthetic mRNA for mRNA vaccine

For designing an mRNA vaccine, the foremost step is the synthetic design of the mRNA. In this regard, a typical mRNA design includes an open reading frame (ORF) encoding the antigen sequence, flanked by 5’ and 3’ untranslated regions (UTRs) accompanying certain artificial modifications to promote efficacy and cellular uptake (26). Additionally, a sophisticated innovation in this technology employs the self-amplifying mRNA (SAM), enabling sustained mRNA augmentation within the host cells. This consequently ensures enhanced production of desired protein aided via the cellular ribosomal machinery. Eventually, the residual mRNA templates are subjected to degradation, hence reducing the potential risk of metabolite toxicity (21). Nonetheless, it is imperative to critically evaluate the potential safety concerns associated with the modifications executed in the vaccine design. Hence, extended research is crucial to ensure the safety profile of the advanced mRNA vaccine platforms.

### 2.2 Identification and optimization of the target antigen

Careful identification and optimization of specific mRNA sequences of target antigens is essential for developing potent mRNA vaccines for cancer. Possible targeted antigens for the development of mRNA vaccine are shown in **Table 2**. In this context, precise sequence designing, followed by efficient synthesis and strategic choice to targeted delivery, is paramount. To address this, multiple strategies are devised for the selection of the target antigen. This mostly involves the utilization of full-length cancer-specific mutant proteins or neoantigens as target TAAs for designing mRNA sequences (71). However, another approach involves a multi-epitope strategy, where immunogenic peptides from multiple TAAs are encoded within the mRNA molecule to stimulate a wide range of immune responses (72). Furthermore, the recent progression in personalized medicine has unlocked new avenues for crafting personalized mRNA vaccines aimed at engineering personalized mRNA sequences to integrate patient-specific mutations in the neoantigens (73). Following the meticulous design of the mRNA sequence, specific nucleotide modifications, such as uridine substitution and the addition of pseudouridine, are introduced to achieve maximal stability, translation efficiency, and precise immunogenicity (26). Additionally, codon optimization is performed using algorithms to prioritize codons readily translated by human cells, thereby maximizing protein expression (26).

**Table 2:** Possible targeted antigens for mRNA vaccine construction for specific cancer type.

Cancer Type	Antigen	Intracellular Location	Expression in Tumor	Reference
Pancreatic	CEA	Cell surface (GPI-linked)	30-60%	(27–29)
	Her2-neu	Transmembrane	7-23%	(30)
	K-Ras	Intracellular	90-95%	(31, 32)
	Mesothelin	Cell surface (GPI-linked)	~100%	(33, 34)
	MUC-1	Transmembrane	90% (Hypo-glycosylation),	(35)
	p53	Intracellular	75%	(36, 37)
	Survivin	Intracellular	68%	(38)
	Telomerase	Intracellular	88%	(39)
	VEGFR	Transmembrane	69%	(40, 41)
Melanoma	Neo-antigens	Varies	Specific to patient	(42)
	MART-1/Tyrosinase	Melanosomes	90%	(43)
	TYRP1, TYRP2	Melanosomes	57-92%	
	Melan-A	Melanosomes	91-97%	(44)
	gp100	Melanosomes	63-90%	(45, 46)
	MAGE-A1	Cytoplasm	51%	(47)
	NY-ESO-1	Cytoplasm	40	(48)
	BAGE, CAGE	Cytoplasm	22%	(49)
	TERT1	Intracellular	44%	(50)
Breast	HER2	Cell Membrane	10-30%	(51, 52)
	MUC1	Cell Membrane	94%	(53)
	CEA	Cell Membrane	43.2%	(54)
	NY-ESO-1	Cytoplasm	42%	(55)
	MAGE-A	Cytoplasm	75%	(56)
Lung	MUC1	Secreted (Produced in Golgi)	86.3%	(57)
	KLRG1	Cytoplasm	-	(58)
	CBFA2T3	Nucleus	-	(59)
	EGFR	Cell Membrane	40-80%	(60, 61)
	KRAS	Cytoplasm	30%	(62)
Colon	CEA	Cytoplasm, Cell membrane	-	(63)
	MUC1	Cell membrane	12%	(64)
	EGFR	Cell membrane	25-82%	(65)
	KRAS	Cell membrane	35-45%	(66)
	Beta-catenin	Cytoplasm, Nucleus	36.9-69.8%	(67)
	Survivin	Cytoplasm	60.8%	(68)
	FRα	Cell membrane	33-44%	(69)
	GUCY2C	Cell membrane	95%	(70)

The *in vitro* transcription (IVT) is a widely applied procedure for synthesizing the mRNA. It utilizes specialized enzymes and optimized reaction conditions for producing large quantities of designed mRNA sequences (74). Additionally, enzymatic amplification, such as rolling circle amplification, is frequently administered for potentially higher mRNA yields (75). The optimization of the carrier vehicles is equally crucial for ensuring the targeted delivery of the vaccine in the human system. In this regard, lipid nanoparticles (LNPs) are usually employed for encapsulation of mRNA to ensure protection, stability, and targeted delivery (76). Moreover, LNPs are carefully designed based on the tumor-specific ligands for efficient delivery and reduced systemic harmful effects. Consequently, a reliable and optimized mRNA can be crafted to induce a robust immune response against cancer. Although, multi-epitope strategy and personalized vaccines offer promising therapeutic avenues against cancer, a careful investigation is crucial to avoid potential immunodominance or off-target effects.

## 3 Modifications in the synthetic mRNA sequence

### 3.1 Chemical modifications

**Figure 1:**
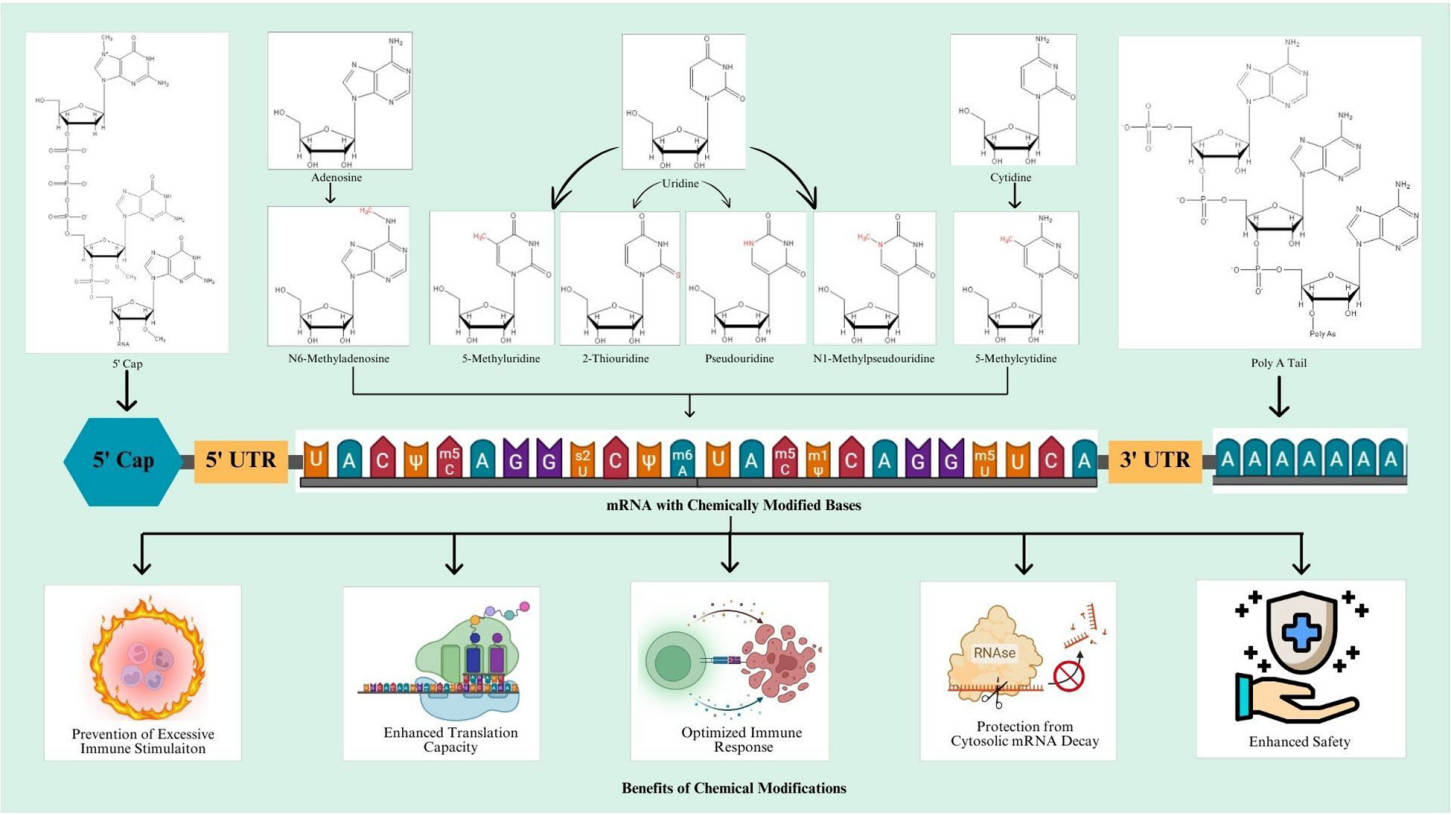
An overview of modified mRNA transcript for mRNA vaccine.

Chemical modifications involve the introduction of chemically modified bases in the mRNA sequence. In this regard, most frequent chemical modifications involve the addition of pseudouridine (Ψ), N1-methyl pseudouridine (m1Ψ), 5-methylcytidine (m5C), N6-methyladenosine (m6A), 2-thiouridine (s2U), and 5-methyluridine (m5U). These modifications render potentially elevated efficiency, stability, and enhanced translational capacity of the resulting mRNA vaccine (77). An overview of modified mRNA with modifications for mRNA vaccine production is shown in **Figure 1**.

#### 3.1.1 Pseudouridine (Ψ)

Pseudouridine (Ψ), an isomer of uridine characterized by the replacement of a characteristic nitrogen-carbon glycosidic bond in the nucleotide with a carbon-carbon covalent bond, is most commonly employed for chemical modifications in the mRNA vaccine (78). This modification is introduced into the mRNA sequence via pseudouridine synthase (PUS) enzyme (79). Thermodynamic analysis revealed the impact of this modification on the overall stability of mRNA. In this regard, the replacement of uridine with Ψ makes the mRNA molecule relatively stable, whereas the level of stabilization varies depending upon the precise location of the modified base within the molecule, the specific base pair subjected to modification, and the orientation of neighboring Watson-Crick base pairs (80). Moreover, the incorporation of Ψ is shown to boost the translational capacity of the mRNA molecule (81). This enhanced translational capacity is achieved by three mechanisms that protect mRNA from decay in the cytosol. Firstly, this modification directly confers resistance to the mRNA molecule against RNase L-mediated degradation (82). Secondly, Ψ modification masks protein kinase R (PKR) activation, ultimately corresponding to a reduction in the PKR-mediated phosphorylation of the eukaryotic translation initiation factor 2 α subunit (eIF2α) (83, 84). Finally, this modification evades the activation of intracellular receptor 2’-5’-oligoadenylate synthase (OAS), which consequently prevents OAS-mediated RNase L activation.

#### 3.1.2 N1-methylpseudouridine (m1Ψ)

The methylated derivative of Ψ, N1-methyl pseudouridine (m1Ψ), is also utilized for replacing the typical mRNA bases. The incorporation of these bases not only renders the resulting mRNA molecule markedly stable but also contributes to their safety by making them to elicit a negligible immune response in the cell, hence reducing their cytotoxic potential (85, 86). Besides, the mRNA molecules harboring modified bases, Ψ and m1Ψ, are found to be inherently inert or minimally reactogenic, hence avoiding excessive immune stimulation (81, 83, 87, 88). Moreover, individual or combinatorial introduction of chemically modified bases has been shown to generate a substantially reduced Toll-Like receptor (TLR) – mediated immunogenicity (89). In this regard, accumulated evidence indicates that m1Ψ outperforms Ψ in achieving elevated protein production and evading TLR3 activation (90). Also, the mRNA modified with m1Ψ escapes TLR7 recognition, hence leading to dismal expression of inflammation-producing genes (91, 92). Furthermore, m1Ψ modification contributes to reduced immune response generation mediated by intracellular Retinoic acid-inducible Gene-I-Like (RIG-I-Like) receptors owing to its ability to alter the mRNA secondary structure (92, 93). Moreover, a direct correlation between m1Ψ-based mRNA modification and enhanced size and proportion of ribosomes is observed (85). This provides a direct link between m1Ψ modification and boosted translational efficiency by developing productive poly-ribosomal interactions as a consequence of rapid initiation and relatively slow progression of translation.

#### 3.1.3 5-methylcytidine (m5C)

5-methylcytidine (m5C), a methylated derivative of cytidine, is frequently observed in multiple RNA species as a natural epigenetic modification (94). This modification is exploited in mRNA vaccine technology as well and confers impressive stability, immunogenicity, efficiency, and sustenance in the cellular environment to the mRNA vaccines (95, 96). In the cellular translation initiation sites, RNA methyltransferases of NSUN and DNMT2 families are found to be substantially enriched for potentially catalyzing the methylation of cytidine bases (97). Similar to the Ψ modification, m5C modification also adds to the mRNA stability and protects it from enzymatic degradation by inducing alterations in the secondary and tertiary structural forms of mRNA (26). A study conducted by Kormann et al. revealed that incorporation of 25% m5C and 25% thiouridine generates 5 times higher protein levels in comparison to the unmodified mRNA (98). Hence, by modulating the level of methylation in the cytidine bases of mRNA sequence via methyltransferases and demethylases, the mRNA vaccines can be efficiently optimized (99).

#### 3.1.4 N6-methyladenosine (m6A)

N6-methyladenosine (m6A), a methylated derivative of adenosine, is another frequent modification observed in naturally existing mRNA transcripts (100). In the cellular environment, this modification regulates mRNA metabolism at various levels, including splicing, nucleus-to-cytosolic export, and stable translation by recruiting reader proteins, including YTHDF1 and YTHDF3 (101). Moreover, with the induction of m6A modification at the specific regions, such as the 5’ UTR or on the bases neighboring the stop codon, the translation efficiency can improve the translation efficiency manifold by active recruitment of translation initiation factors (102). Taken together, this modification significantly boosts mRNA efficacy and safety, and, therefore, serves as a promising tool for improving mRNA-based vaccines.

#### 3.1.5 2-thiouridine (s2U)

2-thiouridine (s2U) is among the novel modifications recently explored for their potential in promoting the activity of mRNA vaccines, and is found to be highly promising (103). Based on the findings of various studies, s2U modification in the IVT mRNA markedly evades recognition from TLR3 and TLR7, hence actively reducing the induction of subsequent inflammatory pathways (104, 105). Furthermore, this modification contributes to mRNA stability by providing resistance against enzymatic degradation. Moreover, the s2U modification promotes mRNA translation efficiency by preventing the activation of PKR, which ultimately results in the inhibition of eIF2α phosphorylation and facilitates protein synthesis (83).

#### 3.1.6 5-methyluridine (m5U)

5-methyluridine (m5U), is another chemically modified base often assessed in the context of mRNA vaccine technology (89). In line with the immunosuppressive mechanisms adopted by the modifications discussed above, m5U also ensures precise immune response generation by evading recognition from TLR3 (90). This promotes mitigation of the innate immune response, hence facilitating efficient delivery of mRNA vaccine into the target cells. Additionally, the incorporation of m5U bases in the mRNA vaccine design renders elevated stability and translation efficiency to the mRNA molecule, consequently improving the vaccine potency (105). After the successful *in vitro* modification of raw mRNA sequence, it is packaged within the delivery vehicles to facilitate the vaccine delivery, protection from degradation, and active cellular uptake (106).

### 3.2 5’ Cap and Poly(A) Tail

5’ Cap and Poly(A) tail mark the key characteristics of eukaryotic mRNA. The 5’ cap, also called “cap 0”, includes an addition of a guanosine molecule methylated at the N7 position to the 5’ end of the first mRNA nucleotide through a 5’,5’-triphosphate bridge (107–109). The addition of the 5’ cap to the mRNA is crucial for appropriate translation initiation followed by ribosome recognition. It protects mRNA from exonuclease-mediated degradation and also facilitates subsequent transcription, polyadenylation, splicing, and mRNA transport from the nucleus to the cytoplasm (110, 111). Several studies have reported that most eukaryotic viral and parasitic mRNAs also harbor a 5’ cap feature (112–114). The addition of a 5’ cap is a well-regulated enzymatic process, and careful monitoring is performed at the cellular level to ensure appropriate capping, as faulty capping can result in premature mRNA decay and compromised translational efficiency. In this regard, the capping machinery involves three specific enzymes, namely RNA triphosphatase, RNA guanylyl transferase, and RNA (guanine-7)-methyltransferase (115). The capping process typically occurs co-transcriptionally, when the pre-mRNA transcript reaches a length of approximately 20-25 nucleotides, which begin to protrude from the RNA exit channel of RNA polymerase II, leading to the addition of cap 0 (116, 117). Following this, further methylation steps are executed involving the addition of methyl groups to the first and/or second transcribed nucleotides, resulting in cap 1 or cap 2, respectively (112). The O-methylation in cap 2 is crucial for demarcating self mRNA from the foreign molecules (118). The 5’ capping marks the natural mechanism of mRNA to cope with the cellular environment and ensure an enhanced level of translation. Hence, it should be considered as a modification mechanism during IVT mRNA synthesis for designing a stable and efficient vaccine.

Additionally, poly(A) tail is another characteristic feature of eukaryotic mRNA, which critically regulates its lifespan (119, 120). It involves the addition of multiple adenosine residues at the 3’ end of the mRNA molecule by a specialized enzyme, Poly(A) Polymerase. This post-transcriptional modification is incorporated into the IVT mRNA vaccine synthesis as well, where the tail length corresponds to overall mRNA stability and efficiency. To achieve this goal, IVT employs various strategies. In this regard, one primarily utilized procedure involves poly(A) tailing of mRNA mediated by recombinant poly(A) polymerase. However, the enzymatic tailing adds poly(A) tails of varying lengths to the mRNA transcripts, thereby resulting in heterogeneous mRNAs concerning the tail lengths. Alternatively, poly(A) tail can be encoded in the template vector designed for *in vitro* transcribing the mRNA molecule to produce synthetic mRNA with uniform tail length. Nevertheless, it is critically challenging to determine the precise duration (121). Regardless of this, IVT attempts to optimize this modification and administers the recombinant poly(A) polymerase to insert modified nucleotides into the tail, preventing poly(A) specific nucleases from deadenylating the poly(A) tail Additionally, poly (A) tailing in IVT mRNA synthesis renders additional benefits to the synthetic mRNA molecule. It has been observed that, for synthetic mRNA, extending the poly (A) tail to 120 bases progressively boosted the level of protein expression, whereas further extension in the tail was not implicated in further uplifting the protein expression levels (98, 122). Therefore, it is imperative to carefully optimize the addition of poly(A) tail in the mRNA molecule for acquiring substantial benefits.

### 3.3 5’ UTR and 3’ UTR

5’ and 3’ UTRs comprise essential regulatory elements of mRNA contributing significantly to the mRNA stability, modulation of complex structures of mRNA, ribosomal recognition, and association of mRNA with the translation machinery (123). Moreover, UTRs can alter the rate of mRNA decay by modulating interactions with various RNA-binding proteins. Studies have reported that skipping the start codon by altering the non-canonical start codons in the 5’ UTR can disrupt translation by various mechanisms, including prevention of stable secondary structure formation, inability to recruit ribosomal machinery and masked codon recognition (124). On the other hand, precise 5’ UTR modification can enhance mRNA stability and translation accuracy. Likewise, 3’ UTR sequences play an equally crucial role in determining mRNA stability and efficiency. In this regard, by imparting considerable stability to the mRNA molecule, 3’ UTR sequences may enable an extended duration of gene expression. In particular, the 3’ UTR of α-globin mRNA is found to harbor discontinuous stretches of pyrimidine-rich regions, which render it extra stablility. This results in the synthesis of messenger ribonucleoprotein α-complex upon recognition of this stretch by cytosolic proteins (125–127). To aid this process, an integral component of α-complex, α-globin poly(C) binding protein (αCP), maintains the attachment of poly(A) binding protein on the poly(A) tail (128). This ultimately renders stability to mRNA by preventing deadenylation of the poly(A) tail. Collectively, UTRs substantially impact mRNA molecules, thereby demanding careful monitoring and optimization of UTR sequences during IVT following comprehensive research and analysis.

### 3.4 Purification of IVT mRNA

During IVT mRNA synthesis, ensuring high-quality production is imperative as it has a direct impact on the efficacy of the vaccine and the subsequent manufacturing steps. Hence, quality control is a critical parameter of IVT mRNA synthesis and should be monitored during various synthesis steps. In this regard, the synthesis process generally includes multiple steps, including a selection of the target antigen sequence, DNA template creation, IVT mRNA synthesis, mRNA purification, and LNP formulation (129). IVT employs several RNA polymerases, including T3, T7, and SP6, for artificially synthesizing high yields of relatively longer mRNA transcripts with length ranging in kilobases (130, 131). However, upon the introduction of modified bases in the IVT mRNA for the first time, the necessity of rigorous purification of the underlying mRNA transcripts for effective immune response generation was emphasized. To address this, various purification techniques were explored. In this regard, size-based mRNA chromatographic purification via high-performance liquid chromatography (HPLC) can eliminate bigger and smaller by-products, including double-stranded RNA, mRNA trances from non-linearized DNA templates, and abortive transcripts (104, 132). This comprehensive purification significantly boosted the output by potentially removing the impurities that may be implicated in negatively stimulating the immune system. Nonetheless, the advancements in the IVT mRNA synthesis and purification techniques have significantly improved the quality and efficacy of mRNA vaccines, the substantial challenges in optimization persist and need to be addressed by thorough research activity.

## 4 Delivering the mRNA and activation of immune response

**Figure 2:**
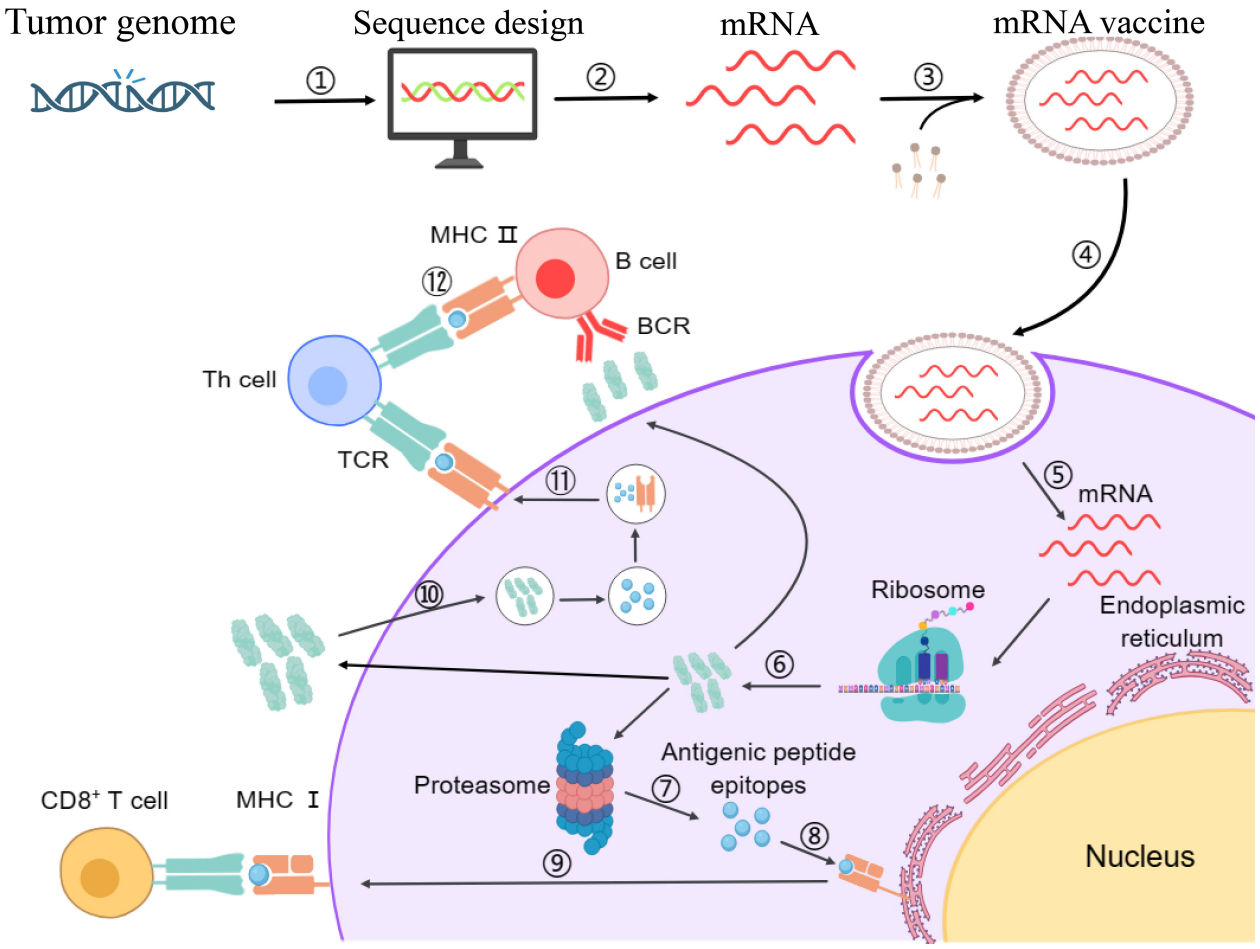
Schematic representation of designing mechanism of mRNA vaccine and its mode of action inside the cells. Step 1: The marked antigen sequence is designed and then introduced into the plasmid DNA vector when the tumor genome has been accomplished. Step 2: Artificial mRNA designed by *in vitro* transcription using the linearized plasmid DNA template is purified. Step 3: The purified mRNA is combined with delivery intermediaries to produce the mRNA vaccine. Step 4: Endocytosis takes the mRNA vaccine up inside the cells. Step 5: Release of the marked mRNA into the cytoplasm. Step 6: The ribosome translates the mRNA into protein. Step 7: The proteasome complex breaks down the protein product into antigenic peptide epitopes. Step 8: In the endoplasmic reticulum, the antigenic epitopes are loaded onto MHC class I molecules. Step 9: MHC class I molecules deliver CD8+ T cells antigenic peptides. Interchangeably, the protein product is designed, captivated by the cell, and then uncovered to an endosomal degradation process in step 10. Step 11: MHC class II molecules present the antigenic fragments to T-helper cells on the cell surface. Step 12: T-helper cells prompt B cells to make antibodies that neutralize target-specific cancer antigens. MHC, main histocompatibility complex, BCR and TCR, T cell and B cell surface receptor. The figure illustrates the overview of modified mRNA for mRNA vaccine production. The structure of mRNA is presented, starting from the 5’ cap, followed by the 5’ untranslated region (UTR), the coding region with chemically modified bases, and the 3’ untranslated region (UTR) with a poly(A) tail. The modifications shown include methylation and isomerization of bases: N6-methyladenosine (m6A), 5-methyluridine (m5U), 2-thiouridine (s2U), pseudouridine (Ψ), N1-methylpseudouridine (m1Ψ), and 5-methylcytidine (m5C), among others. These modifications occur at adenosine, uridine, and cytidine bases, providing specific structural and functional benefits.

Among the core challenges associated with mRNA vaccines, the compromised delivery of mRNA inside the target cell and the subsequent risk of immune rejection are paramount. Additionally, failure to induce the required immune response is another significant limitation. To address these challenges, self-amplifying mRNA technology holds substantial promise. This technology employs an advanced approach involving the incorporation of additional sequences, such as viral RNA replication genes, in the mRNA sequence alongside the target protein sequence (3, 133). Upon expression in the target cell, these additional sequences facilitate replication of the target mRNA, thereby boosting the target protein production (21). The enzymatic process, *In Vitro* Transcription (IVT), remains the gold standard method for synthesizing mRNA from a DNA template within a controlled laboratory setting. In this regard, by utilizing DNA-dependent RNA polymerases, often derived from bacteriophages, IVT eliminates the requirement of an intermediate step involving plasmid DNA, streamlining the overall production process (134). Consequently, the generated mRNA is inherently fragile and necessitates the protection for safe and directed delivery to the target antigen-presenting cells within the body. To overcome this hurdle, specialized LNPs are employed as carriers to encapsulate the designed mRNA molecules (135). The LNPs ensure the safe, efficient, and targeted delivery facilitating the intracellular release of the mRNA (13). Upon the intracellular uptake in the target cells, the mRNA gets translated into the desired protein through the cellular ribosomal machinery (13, 136). The generated proteins are subsequently processed via the proteasomal machinery into shorter antigenic peptides, which are then loaded onto Major Histocompatibility Complex (MHC) molecules in the endoplasmic reticulum (137). MHC-I and MHC-II molecules presenting the antigenic peptides on the cellular surface of APCs engage with the T-cell receptors (TCRs), hence activating the Cytotoxic T-cells (CTLs) and Helper T-cells (HTLs), respectively. Additionally, B-cells recognize and internalize intact antigens through their B-cell receptors (BCRs). After internalization, B-cells process these antigens and present the derived peptides on their MHC class II molecules to helper T-cells. The activation of helper T-cells by this interaction, along with co-stimulatory signals, triggers the differentiation of B cells into antibody-producing plasma cells and memory B cells. Plasma cells then produce neutralizing antibodies that can bind to and neutralize circulating tumor antigens (138). Hence, the mRNA vaccine prompts an immune response within the host and offers a defense against the disruptive influence of the tumor cells (139). **Figure 2** illustrates the entire process of immune activation mediated by mRNA vaccine administration.

## 5 mRNA vaccine delivery systems

### 5.1 Lipid nanoparticles (LNPs) and other delivery platforms

Nucleic acid vaccines are vulnerable to degradation by nucleases in the body, hence requiring specific methods to improve their distribution (140). Moreover, delivering naked mRNA is inefficient due to rapid degradation and poor cellular uptake (141). In this regard, vectors can serve as specialized transport systems that guard nucleic acids from degradation and promote their cellular uptake (142). Gene delivery vectors are broadly classified into two categories: viral and non-viral vectors (143). Viral vectors refer to the carrier systems obtained from the viruses, whereas the non-viral vectors generally integrate artificial particles (144, 145). In terms of eliciting the immune response, the viral vectors have been shown to surpass the non-viral vectors and offer relatively higher efficacy (146). However, non-viral vectors appear comparatively less effective, and potentially less likely to induce immune reactions (147). Moreover, cellular uptake of mRNA remains another significant hurdle owing to the inherent negative charges on both, mRNA and cell membrane, coupled with the relative size of mRNA molecules, and their susceptibility to degradation by ribonucleases in the skin and bloodstream (148). Hence, to address these limitations, strategies for delivering mRNA into the cells have been extensively explored and pinpointed robust delivery carriers, including polymers, peptides, and lipid-based materials (149). In this context, liposomes have emerged as a promising drug delivery system due to their capacity to encapsulate and transport poorly water-soluble drugs (135). Doxil, a liposomal formulation of the antitumor agent doxorubicin, was the initial liposomal drug to receive clinical approval, leading to a shift in the trends in vaccine delivery, with lipid-based nanoparticles being widely utilized in the formulations (150). Moreover, numerous cationic lipid amphiphiles have been developed in recent years and evaluated as carriers of nucleic acids. The primary difference between these cationic lipids and their natural equivalents involves the presence of an additional ionizable (cationic) head group rather than a zwitterionic or a usually anionic head group. Natural and cationic lipids exhibit comparable molecular features, featuring a hydrophobic region consisting of two alkyl chains or cholesterol, and a linker joins their positively charged polar head groups to the hydrophobic moiety. Regarding their vaccine delivery potential, ionizable lipids are a superior choice compared to nonionizable cationic lipids because they are relatively less hazardous, remaining neutral in the circulation and exhibiting a positive charge only upon entering the cell due to pH changes (151). These delivery vehicles can be further optimized to increase the efficacy and safety of mRNA vaccine and thorough research is required to develop more relevant and innovative delivery carriers for mRNA vaccine.

Additionally, for the delivery of mRNA vaccines, many polymer-based vectors have been developed, including poly(l-lysine) (PLL), poly(amido-amine) (PAA), poly (beta amino-esters) (PBAEs), and poly(ethylenimine) (152, 153). However, only PEI has been extensively used in clinical studies. (154). Recently, new lipid-polymer complexes known as charge-altering releasable transporters (CARTs) have been established for the active distribution of mRNA molecules. (155). Moreover, mRNA and cytosine-phosphate-guanine (CpG), a synthetic toll-like receptor-9 agonist, are combined in CARTs to prepare a nanoparticle formulation that effectively transports antigen-coding mRNA to APCs (156). After efficient delivery of the mRNA into the target cells, the mRNA translation, followed by antigen processing and presentation triggers a robust immune response for effectively eradicating existing tumors (157). Specifically, the delivery of antigen-coding mRNA for melanoma immunotherapy, exemplified by the technology behind the Pfizer-BioNTech COVID-19 vaccine, has demonstrated marked effectiveness in clinical trials (158). Furthermore, polymer-based carriers offer a hopeful strategy for mRNA delivery. Nanoparticles made from polymers like poly lactic-co-glycolic acid (PLGA) can provide sustained release of the mRNA and can be potentially combined with adjuvants to further improve the immune response (159). Hence, mRNA encapsulated in a fatty shell offers a promising strategy for facilitating protected mRNA transport and cellular uptake (160). In this regard, mRNA-1273 (Moderna) successfully employed this delivery strategy (161).

### 5.2 Key components of LNP formulation

**Figure 3:**
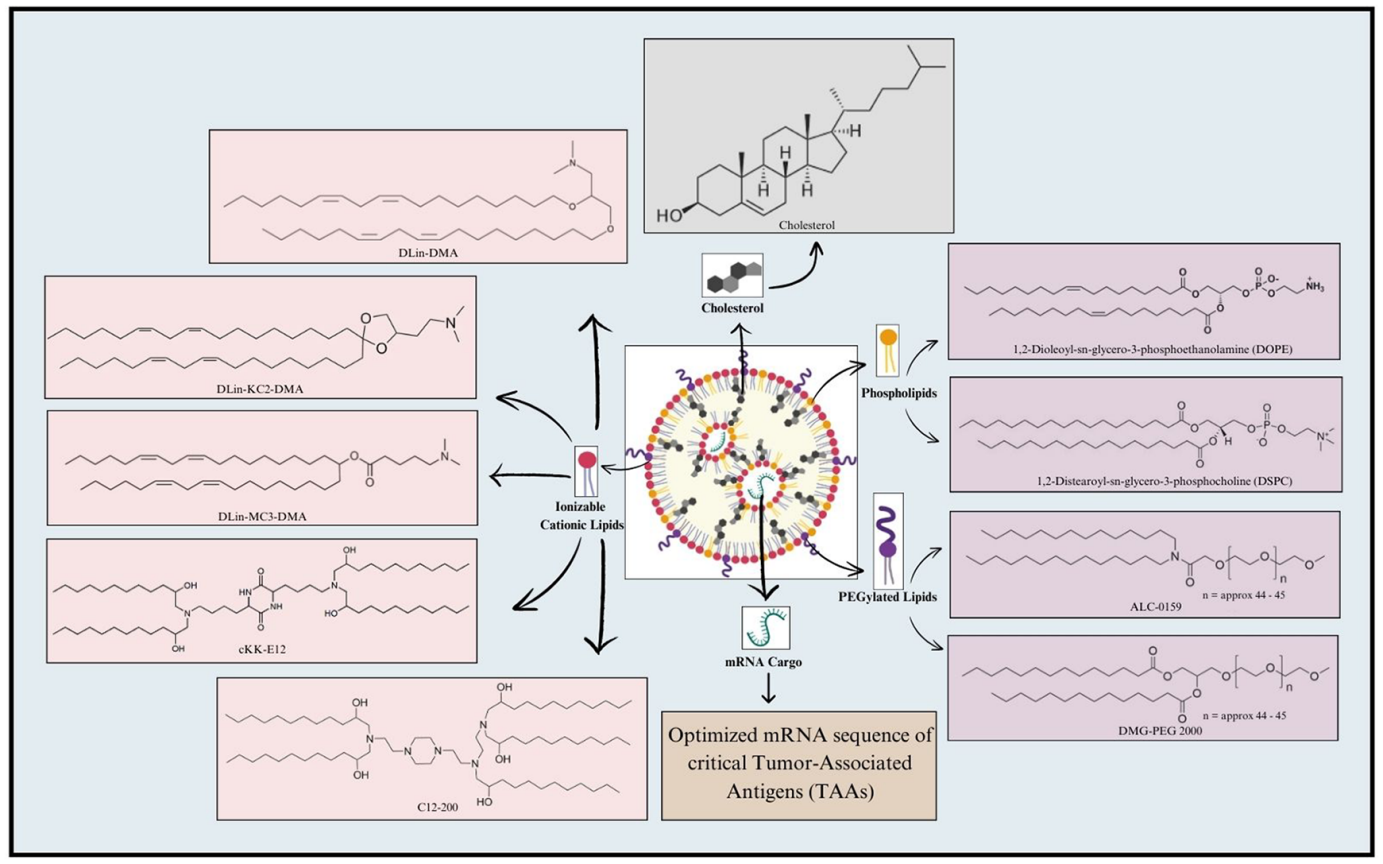
Key components involved in mRNA-LNP formulation. A typical LNP includes four key components, namely Ionizable cationic lipids, Phospholipids, PEGylated lipids, and Cholesterol. Ionizable cationic lipids generally employed for LNP formulation include DLin-DMA; DLin-KC2-DMA; DLin-MC3-DMA; cKK-E12; and C12-200. Phospholipids, such as 1,2-Dioleoyl-sn-glycero-3-phosphoethanolamine (DOPE) and 1,2-Distearoyl-sn-glycero-3-phosphocholine (DSPC), are frequently utilized in this regard. Additionally, PEGylated lipids used for LNP formulation include ALC-0159 and DMG-PEG 2000. Cholesterol is also incorporated in the LNP to maintain its structural integrity. Finally, the mRNA cargo loaded within LNP includes the mRNA sequence of critical genes encoding Tumor-Associated Antigens (TAAs).

LNP formulation typically involves a combination of four major constituents, including ionizable cationic lipids, phospholipids, cholesterol, and PEGylated lipids (162–164). Ionizable lipids play a crucial role in LNP formulations for mRNA delivery (165). Compared to traditional cationic lipid nanoparticles, the ionizable lipid-based LNPs (iLNPs) maintain electrical neutrality under physiological conditions, which helps reduce rapid elimination from the bloodstream and minimize immune system stimulation after intravenous administration (166, 167). Representative ionizable lipids include DLin-DMA, DLin-KC2-DMA (168), and DLin-MC3-DMA (169), synthesized through rational design; C12-200 and cKK-E12 (170), identified via high throughput screening of combinatorial libraries; and next-generation ionizable lipids, such as DLin-MC3-DMA derivative L319 (Alnylam and AlCana Technologies) (171), most of which are biodegradable. The key advantage of ionizable lipids is their ability to undergo protonation at acidic pH, allowing them to interact with the negatively charged mRNA through electrostatic forces (167). Following the cellular uptake and exposure to the acidic endosomal environment, the ionizable lipids become positively charged, hence disrupting the endosomal membrane structure and facilitating the release of mRNA into the cytosol (172). This remarkable alteration in the physiochemical properties of ionizable lipids in a pH-responsive manner offers an impressive potential for mRNA delivery (173), (174). Following this, phospholipids make up the second integral component of LNP formulation, where they significantly contribute to the stability and fluidic nature of LNPs. Precisely, phospholipids, including DOPE and DSPC, generally contribute to as much as 10-30% of the entire lipid composition within the LNP (175). Furthermore, the polarity and degree of unsaturation of the head and tail groups of the phospholipids, respectively, significantly affect the overall design of mRNA-LNPs, and should, therefore, be carefully optimized (167). In this regard, phospholipids harboring head regions equipped with quaternary amino groups coupled with unsaturated fatty acid tails can improve the efficiency of delivery and enhance the potential of mRNA endosomal escape (176). Thirdly, cholesterol serves to be a core component of LNP formulation by contributing significantly to the LNP lipid composition (177). Cholesterol promotes LNP formulation efficiency by various means. In this regard, it offers fluidity for maintaining the structural integrity of LNP during the integration of bulk cargo (178). Moreover, in addition to facilitating mRNA release followed by successful cellular uptake, cholesterol also stabilizes LNP by minimizing the association of proteins on the LNP surface (178). Finally, PEGylated lipids, such as ALC-0159 and DMG-PEG2000, mark the fourth crucial LNP component. Unlike the aforementioned three components, less proportion of PEGylated lipids, generally 0.5-3%, is integrated into the LNPs. PEGylated lipids render ‘stealth’ properties to the LNPs, impressively elevating the *in vivo* circulation duration (179). Moreover, the PEG component provides steric hindrance, preventing aggregation, reducing non-specific uptake, and evading clearance by the mononuclear phagocyte system, thereby enhancing the delivery efficacy of the LNPs (180). Nonetheless, all these elements are essential and contribute to the stability, transfection efficiency, and safety of LPNs (179). Key components of LNP for mRNA vaccine formulation are shown in **Figure 3**.

### 5.3 mRNA-LNP synthesis for mRNA vaccine designing

For mRNA-LNP synthesis, different lipids and mRNA are usually dissolved in the ethanol and acidic aqueous phases (such as pH 4.0 citrate buffer) with a microfluidic device at a volume ratio of 1:3, and the execution of the self-assembly process is enabled. Then, during the formation phase, negatively charged mRNA is allowed to establish electrostatic interactions with the ionizable cationic lipid after the protonation lipid becomes positively charged. Moreover, to stabilize the produced mRNA-LNP, other helper lipids such as cholesterol, phospholipids, and PEGylated lipids self-assemble on them (181). Consequently, the ionizable lipids become uncharged and less hazardous at physiological pH. Following this, the mRNA-LNP solution is buffer exchanged to a neutral pH (182). The latest examples of mRNA vaccines utilizing these LNP components include Moderna COVID-19 vaccine (mRNA-1273) and Pfizer-BioNTech’s COVID-19 vaccine (BNT162b2. Moderna COVID-19 vaccine uses the ionizable lipid SM-102, DSPC, cholesterol, and DMG-PEG2000, whereas Pfizer-BioNTech COVID-19 vaccine employs the ionizable lipid ALC-0315, DSPC, cholesterol, and ALC-0159 (a PEG-linked lipid) (183). Hence, these advanced LNP formulations have undoubtedly played a crucial role in the success and rapid development of mRNA-based COVID-19 vaccines.

Furthermore, when evaluating the efficiency of mRNA-LNP complexes, a critical assessment of the zeta potential is essential, as it significantly affects the delivery efficiency and biodistribution of LNPs (184). In this regard, LNPs undergo a transition from a net positive charge at low pH to a negative charge at high pH, as detected by the broad ZP titration curve (185). This charge transition is important for the endosomal escape of the mRNA payload. At low endosomal pH, the positively charged LNP can form ion pairs with anionic endosomal phospholipids, destabilizing the endosomal membrane and releasing the mRNA. Additionally, the breadth of the ZP titration curve (over ~4 pH units) indicates that the LNPs maintain a positive charge across the entire endosomal pH range, facilitating the membrane disruption and cytosolic delivery of mRNA (185, 186). The net charge of the LNPs, as measured by ZP, also determines the *in vivo* biodistribution and targeting of LNPs (187). In this regard, cationic LNPs tend to accumulate in the lungs after intravenous (IV) administration, whereas, reducing the cationic lipid content for crafting more negatively charged LNPs leads to preferential targeting of the spleen after IV injection (188). Additionally, for intramuscular (IM) administration, more negatively charged LNPs exhibit higher off-target expression in the liver compared to less negatively charged formulations (185). The positive charge of LNPs facilitates their cellular uptake by interacting with negatively charged heparin sulfate proteoglycans on the cell membrane. These positively charged LNPs also demonstrated correlated potency for intramuscular (IM) administration in mice (189). Moreover, LNPs with a positive charge throughout the endosomal pH range are more efficient for *in vitro* mRNA transfection in HEK293 cells (185). In addition, the positive charge is beneficial for IM delivery, likely due to interactions with negatively charged proteoglycans in the extracellular matrix of muscle tissue. Conversely, for intravascular (IV) administration, negatively charged LNPs are favored for hepatocyte targeting (190) The negative charge facilitates passive ApoE-mediated targeting and binding in the bloodstream (191). Nonetheless, positively charged LNPs have potential applications in mRNA delivery for *in vivo* muscle gene expression and IV administration, as they can target the spleen (192).

### 5.4 Integration of novel biocompatible nano-carriers for mRNA vaccine designing

Numerous newly synthesized materials are being utilized to create PEG-free nano-carriers to boost biocompatibility. A recently developed material, tB-UC18 (comprising a benzene-ring scaffold and three unsaturated lipid tails), has been employed to self-assemble with the aid of 1,2-dioleoyl-sn-glycerol-3-phosphoethanolamine, forming lipid-like nanoassemblies (LLNs) (193). These LLNs have demonstrated thermostability and resistance to nuclease degradation. Moreover, when mRNA was encapsulated in LLNs, the delivery system maintained thermal stability for at least two weeks without PEG lipids. In this regard, a newly developed PEG-free mRNA vaccine, PFTCmvac, exhibited a marked immunogenic potential. The vaccine was capable of generating broad-spectrum adaptive immunity with negligible side effects in mice and evasion of complement system activation in the human serum. This strategy can be further exploited in the mRNA vaccine technology for developing PEG-free nano-carriers (194).

Recently, a novel drug delivery strategy, namely Lipid-polymer hybrid nanoparticles (LPHNPs), has emerged that integrates the beneficial aspects of liposome carriers coupled with biodegradation polymeric nanoparticles (195). This carrier features an innovative structure encompassing three components, including a polymeric core, a surrounding lipid monolayer, and an outermost PEGylated layer (196). The LPHNP formulation involves a sophisticated combination of biodegradable polymer, zwitterionic phospholipids, ionizable lipids, PEG-lipids, and cholesterol (196). The LPHNP-based hybrid nano-carrier system offers striking benefits, including improved transfection properties, targeted mRNA delivery, enhanced stability, and upgraded mRNA release kinetics (174, 197). Moreover, the biocompatible nature of LPHNPs renders them significant resistance against degradation and boosts their ability to facilitate mRNA encapsulation, stability, and cellular uptake (174, 196, 198). Additionally, their optimized hydrodynamic parameters including sizes within the 200-250 nm range further add to their benefits (199). Hence, these nanoparticles significantly widen the therapeutic window of mRNA vaccines in combating diverse cancers and genetic disorders (197, 198).

Taken together, the incorporation of innovative delivery methods can substantially revolutionize the mRNA vaccine technology by offering better optimization parameters. In addition, vaccine administration parameters should also be carefully optimized. In this regard, the administration of SARS-CoV-2 mRNA-LNP via a needle-free injection method ensured a minimally invasive procedure, while achieving enhanced immunogenicity compared to conventional needle-based injection methods (200). In the future, further optimizations and modifications can be executed to improve delivery, stability, targeting, immune stimulation, cost-effectiveness, and yield for wider applicability of mRNA vaccines in clinical set-up (186).

## 6 Mechanism of mRNA endosomal escape from mRNA -LNP complex

The efficient intracellular delivery of mRNA payloads is a crucial requirement for the success of mRNA-based therapies (H. Liu et al.). After cellular internalization, mRNA-loaded lipid nanoparticles (LNPs) must escape the endosomal compartment and release the mRNA into the cytoplasm, where it can then be translated into the desired protein (201, 202). In this regard, two primary mechanisms have been proposed, including “membrane destabilization” and “proton sponge effect”.

Firstly, membrane destabilization involves the protonation of ionizable lipids in the acidic pH of endosomes, followed by interactions with anionic lipids on the endosomal membrane. This induces a non-bilayer, hexagonal structure that can disrupt the membrane and release the mRNA payload. (168). Various studies have demonstrated that a pKa range of 6.2 to 6.5 is optimal for effective *in vivo* silencing in hepatocytes, with the highest potency observed at a pKa of 6.44 (169). Moreover, the optimal pKa for protein expression from mRNA-LNPs can be altered by route of administration. In this regard, a study showed that vaccine administration via the intravenous route exhibited a pKa lower than that required for protein expression compared to the administration via the intramuscular route (185, 189). Secondly, the ‘proton sponge effect’ functions by the buffering capacity of LNPs to activate the proton pumps, increasing the membrane potential, This leads to the influx of chloride ions, increasing the osmotic pressure and causing the endosome to swell and burst, thereby releasing the mRNA (203).

After the successful release of mRNA from the endosome and into the cytoplasm, it interacts with the cellular translation machinery to be expressed as a functional protein (204). This process involves ribosome recognition in which the mRNA must be in an unstructured, single-stranded form that can be accessed by the ribosome, the cellular organelle responsible for protein synthesis. Certain mRNA design elements, such as the 5’ cap structure and 3’ poly(A) tail, can enhance mRNA stability and promote efficient translation initiation by the ribosome. Moreover, the mRNA sequence and codon optimization can also impact translation efficiency. Therefore, by integrating the endosomal escape mechanisms and the subsequent mRNA translation process, mRNA-based vaccines can effectively deliver the genetic payload and express the target proteins within the cell.

## 7 Clinical efficacy of lipid-based nanoparticles and other nanoparticles for mRNA delivery

With the recent innovations in the delivery methods, a lot of new approaches are employed to increase the efficacy of mRNA-based therapeutics for cancer treatment. Recently, Miao et al. created a range of ionizable lipid-like materials and validated the best candidate formulations as mRNA delivery vehicles for vaccination against cancer (205). These LNP formulations were used to deliver ovalbumin (OVA) mRNA to the OVA-expressing B16F10 mouse melanoma model. The egg white protein OVA is commonly used as a model antigen owing to its potential of enhanced cytotoxic lymphocyte-based recognition of neoantigens (206). After the initial two doses, the LNP-based mRNA vaccines significantly suppressed tumor growth and produced a robust antigen-specific cytotoxic T-cell response. Additionally, rather than activating TLRs, these LNP formulations stimulated adaptive immune cells via the stimulator of interferon genes (STING) pathway, which resulted in strong antigen expression, and local production of pro-inflammatory cytokines (207). Furthermore, recently a formulation was designed based on a common tumor suppressor gene, phosphatase, and tensin homolog deleted on chromosome ten (PTEN), which is mutated or deleted in a variety of human cancers. In this regard, the lipid materials G0-C14, polylactic-co-glycolic acid (PLGA), and lipid-PEG were used to synthesize mRNA-LNP, and PTEN was reintroduced into cancer cells to restore its ability to serve as a tumor suppressor (208). Another research, focused to craft and refine a platform of mRNA nanoparticles, targeted at C-X-C-Motif Receptor 4 (CXCR4) to effectively increase p53 expression in models of hepatic cell carcinoma (HCC). They combined anti-PD-1 immunotherapy with CXCR4-targeting p53 mRNA nanoparticles, which revealed the massive potential of this combination strategy in enhancing the liver cancer anti-tumor immune response and suppressing tumor growth (209). The researchers demonstrate immense confidence in the potential of this therapy to effectively treat immunosuppressive cancers, alongside liver cancer, and are eager to apply the potential findings from animal models to human clinical trials.

Recently, lipid calcium phosphate nanoparticles (LCPs) were used in the development of a vaccine against melanoma to deliver two essential components, including siRNA directed against the immunological checkpoint PD-L1 and mRNA encoding the tumor antigen TRP-2. Following their loading onto the dendritic cells (DCs), these LCPs were injected into mice. The outcomes demonstrated the effective transport of siRNA and mRNA to DCs in the lymph nodes, which prompted CD8+ T cells to mount a targeted immune response against TRP-2, which significantly slowed down tumor development in mice models (157). Overall, the promising results indicate that LNP-based mRNA vaccines have the potential to become an effective strategy for cancer immunotherapy. To completely comprehend the mechanics and long-term impacts of these formulations, additional meticulous research and critical evaluation are required.

## 8 mRNA vaccine for specific cancer types

Therapeutic mRNA cancer vaccines have emerged as a promising novel approach to cancer immunotherapy (25), offering high specificity, better efficacy, and fewer side effects compared to traditional therapeutic strategies (210). In this regard, multiple therapeutic mRNA cancer vaccines are subjected to stringent evaluation in preclinical and clinical trials, with promising early-phase results (211). Recently, preclinical research has been conducted on mRNA melanoma vaccines using orthotopic mice models, where B16F10 melanoma cells were employed as an experimental cell line. The findings revealed strong stimulation of T cells immune response in the mice model upon vaccine administration, suggesting the promising effectiveness of the vaccine in clinical settings (212). Moreover, immune checkpoint inhibitors, including monoclonal antibodies to counter CTLA-4, PD-1, and PD-L1, have been permitted for therapeutic use. These drugs are explicitly intended to address melanoma, suggesting a synergistic or combined mRNA vaccine approach against melanoma, which can further enhance both efficacy and strength of immune response (213). Furthermore, an mRNA vaccine designed by integrating several MHC classes I and II-restricted neoepitopes produced from B16F10 melanoma cells was shown to be efficacious by Kreiter et al. in 2015 (214). In a mouse model, this vaccination effectively generated strong tumor-specific CD4+ and CD8+ T cell responses, which resulted in considerable tumor rejection (60–80% survival). Expanding on this achievement, Chen et al. created a new formulation of lipid nanoparticles (113-O12B) that demonstrated improved lymph node targeting compared to conventional LNP formulations (215). Notably, an mRNA vaccine expressing a Trp2 180-188-specific epitope was more effective owing to the enhanced delivery method, resulting in a 40% complete response rate in mice challenged with melanoma (216). The injectable vaccination Melanoma FixVac BNT111, administered by liposomal RNA (RNA-LPX), was the subject of a recent clinical trial (Lipo-MERIT, NCT02410733) (217). This first-ever human Phase I trial assessed the vaccine safety and effectiveness in patients with metastatic melanoma. Four frequently occurring, non-mutated tumor-associated antigens in melanoma were targeted by melanoma FixVac BNT111. The trial’s initial dose-escalation phase established the safety and tolerability of the vaccination. Also, encouraging findings from an intermediate analysis were found (218).

Recently, a novel adjuvant therapy strategy for melanoma combining anti-PD-1 medication with a personalized mRNA-based cancer vaccine has been constructed. The neoepitopes have been shown to efficiently induce robust anticancer immunity *in vivo* (219). In the KEYNOTE-942 stage II test, patients with resected high-risk stage IB–IV melanoma were randomized to receive either pembrolizumab alone or the mRNA-4157 vaccine, leading to enhanced immune response generation (220). Another study examined the immunogenic potential of GM-GSF as an adjuvant along with the mRNA vaccination encoding six melanoma-specific TAA genes in a phase I/II clinical trial involving 21 melanoma patients in 2009. The data showed that immunization significantly reduced the number of immunosuppressive cells (Foxp3+/CD4+T cells) without creating any adverse side effects (221). Furthermore, an mRNA vaccine, namely Lipo-MERIT, combining PD-L1 with four TAAs, was introduced to melanoma. Consequently, it effectively triggered IFN-γ production and encouraged the recruitment of T cells specific to the antigen (NCT02410733) (222). Afterward, recently developed lymph node-targeted LNP-based mRNA vaccination enhanced antitumor immunity and produced robust CD8+ T cell responses in B16F10 melanoma-bearing mice (215). Additionally, Christian et al. demonstrated high efficacy in mouse tumor models by encoding a variety of cytokines, including GM-CSF, IL-15, interleukin-12 (IL-12) single chain, and interferon-α (IFN-α), in an mRNA combination through multiple intratumoral injections against melanoma. Furthermore, when the tumor growth was effectively controlled, the majority of them completely disappeared. This was further linked to several factors, including the formation of immunological memory, amplified granzyme B+, T-cell infiltration, intratumoral IFN-γ induction, systemic antigen-specific T-cell growth, and anticancer activity of mRNAs encoding four different cytokines. This defensive response was also evident in metastatic areas external to the initial tissue, where mRNA-encoded cytokines are generated *in vivo* (215). This offers a promising hope for combining cytokines with mRNA vaccine as an adjuvant therapy for melanoma.

Recently, a phase II clinical trial of the GP2 peptide-based vaccine, under the vaccine ID NCT00524277, showed groundbreaking outcomes of a 100% 5-year survival rate in patients with HER2+ breast cancer (223). Although no breast cancer vaccine has yet been permitted for either treatment or inhibition, this evokes a general interest that breast cancer mRNA vaccine would have great implications in significantly improving the survival rate. Another ongoing clinical trial (NCT03289962) targeting TNBC with RO7198457 (individualized mRNA vaccination) + atezolizumab (anti-PD-L1) has shown that tumors overexpressing HER2 show less response to immune checkpoint blockade (224). However, by revitalizing T-cells, HER2-specific mRNA vaccination may enhance the sensitivity of cancer cells to immune checkpoint inhibitors (225). Moreover, research has revealed that the mRNA vaccination and the anti-CTLA-4 monoclonal antibody can work in conjunction to produce a strong CTL response against TNBC (226). Moreover, another study has revealed the effectiveness of mannose-modified lipid-core nanoparticles (LCP) in delivering MUC1 TAA encoding mRNA to the dendritic cells, triggering a robust immune response against TNBC cells (227). In addition, new research investigated the efficacy of VRP-HER2, a viral-based HER2 RNA vaccine, in a mouse model. Based on the findings, a considerable induction of HER2-specific T-cells mediated by VRP-HER2 was revealed, substantially restricting tumor growth. The HER2-specific T-cells induced by this vaccination were CD8+ T-cells capable of expressing perforin, which has been previously reported to be implicated in promoting disease-free survival in breast cancer patients (228). Additionally, clinical trials of TriMix vaccine (NCT03788083) are in progress to evaluate the safety and efficacy parameters of intratumoral mRNA vaccine against breast cancer (229). Besides this, individualized mRNA neoantigen vaccine (iNeST) is also under assessment in amalgamation with added lipoplex-formulated mRNA-producing TAAs (BNT114), and RNA encrypting p53 in patients with TNBC (NCT02316457). Currently, CARvac, an mRNA lipoplex vaccine encoding claudin-6 protein (CLDN6), is under inspection in a phase 1/2 clinical trial (NCT04503278) for patients with advanced solid CLDN6-positive tumors. Moreover, this vaccine can also be combined with mRNA technology to further boost its efficacy. To improve CAR T-cell treatment, BNT211, an autologous CLDN6, and CARVac are simultaneously injected intravenously. Early findings showed that four (57%) of the seven evaluable patients treated with a combination of CARVac and CLDN6 CAR T-cell therapy exhibited a partial response, while one patient (14%) remained stable at the 6-week evaluation (230, 231). BioNTech is currently developing BNT114, a pre-made set of common cancer antigens. Conversely, BNT-122 is a personalized vaccine targeting a patient-specific tumor mutation (211). Following this, the phase I clinical trial of AVX901, an amplifying mRNA vaccine based on a self-amplifying VEEV vector articulating HER2, has been completed. Next, phase II clinical investigation aims at assessing the efficacy of AVX901 in combination with pembrolizumab (232). Moreover, NCT01837602, a phase 0 clinical trial, is now investigating the safety and feasibility of anti-cMet chimeric antigen receptor T cells (CAR-T cells) to treat patients with metastatic breast cancer (233). TNBC patients have undergone evaluations of the safety, immunogenicity, and effectiveness of a customized mRNA vaccine made with neoantigen liposomes (NCT02316457), where the preliminary results reflect a strong generation of poly-epitopic T-cell response (234). Recently, a stage 1 clinical trial of a custom-made mRNA vaccine, autogenous cevumeran, displayed that it can initiate improved immune responses in 50% of PDAC patients after medical resection, which increases the hope of treating this deadly disease. In this regard, the vaccine design is equipped with 20 neoantigens and is transported using LNPs via circulatory injection in amalgamation with chemotherapy and immune checkpoint therapy (235). The Phase I clinical trial results confirmed the clinical benefits for patients with surgically resected PDAC (NCT04161755) and the ability of autogene cevumeran to expand neoantigen-specific and functional CD8+ T cells (236). Moreover, MUC1 is distinguishably an overexpressed TAA in pancreatic cancer, making it a superlative target of therapeutic design (237). Furthermore, the high frequency of KRAS point mutations in pancreatic cancer suggests it to be a potential target for therapeutic strategy against pancreatic cancer (238). In this regard, a personalized mRNA vaccine can be constructed by targeting patient-specific KRAS mutations. Moreover, an ongoing medical trial has recruited patients for appraisal of the safety and acceptability of mRNA5671/V941, either unaccompanied or in grouping with pembrolizumab, for the treatment of progressive or metastatic pancreatic cancer (NCT03948763) (107). Another mRNA-based vaccine trial is undergoing in clinical settings to assess the protection and effectiveness of progressive adjuvant atezolizumab (Genentech) and autogene cevumeran in patients with PDAC (235).

Following this, another clinical trial to appraise the protection and worth of a customized mRNA neoantigen vaccine (iNeST), in grouping with mFOLFIRINOX, a first line of therapeutic combination for PDAC, in patients with surgically resectable PDAC is in progress. The main aim for designing this vaccine is to intensify neoantigen-specific T-cells repressed by PD-1 signaling and major naive T-cells in response to vaccine neoantigens. The iNeST has been shown to be considerably safe with marked feasibility and dismal side effects in patients. It has also been shown to produce significant neoantigen-specific T-cells in 50% of unselected patients with resectable PDAC. Furthermore, vaccine-induced T-cells are shown to be resilient and can last up to two years after receiving mFOLFIRINOX treatment (235). Additional ongoing clinical trials for solid tumors, including PDAC, are mounting on Phase 2 with the reference IDs: NCT03289962 and NCT04161755 (236). Afterward, mRNA-4650, an mRNA vaccine targeting KRAS mutations, developed by Moderna and Merck, was used in amalgamation with or without pembrolizumab for the treatment of pancreatic carcinoma to create adjuvant or combinational therapy. The result of the therapy evaluated that the LNP distribution system for mRNA-4650 was well endured and provoked an anti-tumor immune response (19). Likewise, hTERT vaccination shows promise in inducing an immune response against cancer, with a case study demonstrating complete remission in a pancreatic cancer patient following DC vaccination with hTERT mRNA. This successful treatment also identified novel epitopes that can be incorporated into future hTERT vaccines for broader application (239). The latest mRNA-based and peptide-based combinational vaccines have been constructed to incorporate specific antigens and mRNA. In this regard, one such vaccine, mRNA-5671/V941, targets four KRAS mutations, including G12D-, G12V-, G13D-, and G12C-, is tested in combination with pembrolizumab in stage I experimental trial for patients with KRAS-mutant advanced colorectal cancer under the clinical reference ID of NCT03948763 (240–242). During the trials, the vaccine was administered intramuscularly in LNPs for nine cycles every three weeks. Consequently, significant antitumor response was observed with marked tolerance of the formulation *in vivo*. Upon translation and processing of the antigen protein, peptide epitopes were presented via MHCs and enabled the generation of T-cell mediated immune responses (6). Additionally, two NSCLC-specific mRNA vaccines, CV9201 (NCT00923312) and CV9202 (NCT01915524) were evaluated in clinical trials to assess their therapeutic potential against lung cancer in combination with checkpoint inhibitors. CV9201 targets five TAAs specifically linked to NSCLC, whereas CV9202 is a self-adjuvating mRNA vaccine that targets six antigens commonly expressed in NSCLC (NY-ESO-1, MAGEC1, MAGEC2, 5 T4, survivin, and MUC1) (243). By facilitating antigen expression and stimulating TLR7/8 and intracellular RNA sensors, they aim to activate immune cells against NSCLC cells (244). Based on the initial results, the vaccines were shown to be safe and efficient in producing antigen-specific T-cell immune responses in 63% (for CV9201) and 84% (for CV9202) of individuals. Nevertheless, neither progression-free survival nor overall survival was observed to be enhanced by CV9201 (245, 246). Moreover, a vaccination trial, NCT00004604, is carried out to evaluate the safety and dose-limiting toxicity of a vaccine including DCs injected with CEA-encoding mRNA. Phase I data indicated that, while all patients felt malaise and subcutaneous nodules at the injection site, no major toxicities were noted. As a result, it was established that giving CEA mRNA-transfected DCs was safe (247, 248). Recently, multi-epitope neoantigen was synthesized as a KRAS mRNA vaccine, mRNA-1521, that wholly covered all predominant KRAS mutations to attain a broad-spectrum immune response. Following this, immune response and anti-tumor effectiveness were measured in the colon cancer Balb/c mice model. It was noted that prophylactic immunization with mRNA-1521 pointedly repressed tumor growth in the mouse model of colon cancer. The repressive effects of the vaccine were additionally improved upon the combination with anti-PD-1 antibodies (249). mRNA-1521 also provoked specific T and B cell responses vital for anti-cancer immunotherapy (249). A single intratumorally administered injection of (murine) IL-12 mRNA-LNP, under the vaccine ID (NCT03946800), produced a tumor clearance rate of approximately 86% in MC38 mouse models with colon adenocarcinoma, and almost all of the mice were resistant to tumor recurrence (250). A vaccination based on OMV-LL-mRNA has been shown to dramatically slow the growth of melanoma and induce 37.5% full remission in a colon cancer model. After 60 days, OMV-LL-mRNA markedly protected the mice from tumor challenges by inducing a long-term immunological memory (251). Furthermore, the mRNA cancer vaccination expressing the MC38 neoantigen successfully suppressed MC38 colon cancer mice and prevented tumor recurrence when given in concert with immune checkpoint blockade therapy (152, 252). Finally, phase I/II clinical trials are currently ongoing to evaluate the therapeutic potential of a KRAS-targeting mRNA vaccine, ELI-002 against operable colorectal cancer (242). The successful findings revealed by these clinical trials boost the confidence of researchers in the clinical applicability of mRNA vaccines against cancers. Nonetheless, extended evaluation is imperative to unfold the long-term effects of these vaccines before their utilization in cancer therapeutics. mRNA vaccine for different cancer types is shown in **Table 3**.

**Table 3:** mRNA vaccine for different cancer types.

Trail identifier	Targeted malignancy	Vaccine type	Target antigens	Combination therapy	Route of administration	Results or recruitment status	Sponsor/ Collaborators
NCT04335890	Uveal Melanoma	Personalized dendritic cell (DC)	Autologous tumour RNA with gp100, tyrosinase, PRAME, MAGE-A3, IDO, and different driver mutations	V940 (mRNA-4157) + KEYTRUDA	Intravenous infusion	Ongoing Phase 3 trial	Hasumi International Research Foundation
NCT01456104	Melanoma	Langerhans-type dendritic cell vaccine	Murine tyrosinase-related proteins	Langerhans-type dendritic cells + checkpoint inhibitor antibody	Intravenous injection	Ongoing Phase 1 trial	Memorial Sloan Kettering Cancer Center
NCT02285413	Melanoma	Autologous monocyte-derived dendritic cell (DC) vaccine	gp100, tyrosinase (melanoma-associated antigens)	Standard chemotherapy	Intradermal and intravenous	Completed Phase 2 randomized trial	–
NCT01302496	Previously treated unresectable stage III or IV melanoma	Autologous TriMix-DC therapeutic vaccine	MAGE-A3, MAGE-C2, tyrosinase, and gp100	TriMix-DC vaccine combined with ipilimumab (anti-CTLA-4 monoclonal antibody)	Intravenous and intradermal	6-month disease control rate of 51%, overall tumor response rate of 38% (8 complete responses and 7 partial responses). Some patients remained disease-free for over 5 years	Universitair Ziekenhuis Brussel, with Bart Neyns as the principal investigator
NCT01983748	Uveal melanoma	Autologous tumour RNA vaccine	autologous tumor RNA antigens.	None	Intravenous	Ongoing, no results reported	University Hospital Erlangen, Germany
NCT01302496	Previously Treated, Unresectable Stage III or IV Melanoma	Autologous TriMix-DC Therapeutic Vaccine	MAGE-A3, MAGE-C2, tyrosinase, gp100	Ipilimumab	Intradermal (ID) and Intravenous (IV)	6-month disease control rate of 51%, overall tumor response rate of 38% (8 complete responses and 7 partial responses). Some patients remained disease-free for over 5 years.	Universitair Ziekenhuis Brussel, Vrije Universiteit Brussel
NCT01676779	Unresectable AJCC stage III or IV melanoma	Autologous monocyte-derived dendritic cells (DCs) electroporated with synthetic mRNA	MAGE-A3, MAGE-C2, tyrosinase, and gp100	TriMixDC-MEL vaccine alone and in combination with the CTLA-4 inhibitor ipilimuma	Intravenous (i.v.) and intradermal (i.d.)	TriMixDC-MEL was well-tolerated and may improve the 1-year disease-free survival rate compared to the control group	Melanoma
NCT02410733	Advanced melanoma, specifically stage IIIB-C and stage IV	Tetravalent RNA-lipoplex cancer vaccine	MAGE-A3, NY-ESO-1, tyrosinase, and TPTE	Melanoma FixVac vaccine in combination with anti-PD1 therapy	Intravenous	Recruiting, evaluating safety and tolerability of intravenous administration of Lipo-MERIT vaccine. The melanoma FixVac vaccine, alone and with anti-PD1 therapy, showed durable objective responses in checkpoint inhibitor-experienced patients with advanced melanoma	BioNTech
NCT04526899	Melanoma	Tetravalent RNA-lipoplex cancer vaccine	NY-ESO-1, tyrosinase, MAGE-A3, and TPTE	With cemiplimab	Intravenous	Ongoing Phase 2 clinical trial	BioNTech
NCT03815058	Advanced melanoma	Personalized cancer vaccine encoding individual tumor mutations (BNT122)	Personalized set of tumor mutations	With pembrolizumab	Intravenous	Ongoing Phase 2 clinical trial	Genentech
NCT03394937	Resected melanoma (stages IIc, III, and IV)	Non-formulated (naked)	CD40L, CD70, caTLR4; tyrosinase, gp100, MAGE-A3, MAGE-C2, and PRAME	None	Not reported	Recruiting	Not reported
NCT03897881	Resected high-risk cutaneous melanoma (stage IIIB-IV)	Personalized cancer vaccine mRNA-4157	Up to 34 patient-specific mutated neoepitopes	Pembrolizumab	mRNA-4157: Intramuscular - Pembrolizumab: Intravenous	Recruiting	ModernaTX, Inc.
NCT01526473	HER2+ Breast Cancer	SAM (self-amplifying mRNA) vaccine (AVX901)	HER2	None	Intramuscular	Completed, Safety and Toxicities	- H. Kim Lyerly - Susan G. Komen Breast Cancer Foundation - Duke University
NCT03632941	HER2+ breast cancer	SAM vaccine (AVX901)	Tumor-associated antigens (TAAs)	HER2 vaccine + Keytruda (pembrolizumab)	Intramuscular	Active, not recruiting	Duke University
NCT00978913	Breast Cancer, Malignant Melanoma	Autologous Dendritic Cell Vaccine	Survivin, p53, HER2 (ERBB2)	Cyclophosphamide	Intradermal (i.d.)	Completed, Safety and Toxicities	Inge Marie Svane
NCT00004604	Metastatic Cancer (including Colon Cancer and Adenocarcinoma)	CEA RNA-Pulsed Dendritic Cell Vaccine	CEA, MUC-1 α-gal Epitope	Gemcitabine Chemotherapy	Not Specified	Active, Not Recruiting	Duke University
NCT01291420.	dvanced breast cancer, malignant mesothelioma, glioblastoma multiforme (Grade IV), sarcomas, colorectal tumors, and rare tumors	autologous dendritic cell (DC) vaccine	Wilms' Tumor Gene (WT1) mRNA	With TMZ (temozolomide)	Not specified	Active, Not Recruiting	Antwerp University Hospital, Center for Cellular Therapy and Regenerative Medicine
NCT02316457	Triple-negative breast cancer (TNBC)	Liposome-formulated intravenous RNA vaccine	Neoantigens based on next-generation sequencing screening	None mentioned	Intravenous	Active, Not Recruiting	BioNTech SE
NCT03739931	Advanced solid tumors	In situ vaccine (ISV) with mRNA-2752 (Human OX40L, IL-23, and IL-36γ)	Tumor-associated antigens, with a focus on neoantigens	Intratumoral injections of mRNA-2752 alone and in combination with intravenously administered durvalumab	Intratumoral injection	Phase 1, open-label, multicenter study actively recruiting participants, with Dose Escalation, Dose Confirmation, and Dose Expansion Parts	ModernaTX, Inc.
NCT03788083	Early-stage Breast Cancer	ISV	Trimix mRNA encoding CD40L, CD70, acTLR4, administered using synthetic naked mRNA	Monotherapy, no combination therapy	Intratumoral	Recruiting, results not yet available	Universitair Ziekenhuis Brusse, eTheRNA immunotherapies
NCT01995708	Multiple Myeloma	Autologous CT7/MAGE-A3/WT1 mRNA-Electroporated Langerhans-Type Dendritic Cells	CT7, MAGE-A3, WT1	Standard treatment for multiple myeloma patients undergoing autologous stem cell transplantation	Intradermal	Completed Phase 1 study	Memorial Sloan Kettering Cancer Center
NCT03908671	Esophageal cancer, Gastric adenocarcinoma, Pancreatic adenocarcinoma, Colorectal adenocarcinoma	Personalized mRNA Tumor Vaccine	RNA, Solid tumors	Personalized mRNA Tumor Vaccine	Subcutaneous	Recruiting	Stemirna Therapeutics
NCT03468244	Gastric adenocarcinoma, Pancreatic adenocarcinoma, Colorectal adenocarcinoma	mRNA neoantigen vaccination	Mutated neoantigens, Oncoviruses, Endogenous retroviral elements (HERVs), Unconventional antigens	Personalized mRNA vaccine	Subcutaneous	Not specified	Changhai Hospital, other institutions (OIs)
NCT04163094	Ovarian cancer	BNT115 (ovarian cancer tumor-associated antigens)	Not applicable	Carboplatin and paclitaxel	Intravenous	Recruiting	University Medical Center Groningen (Groningen, Netherlands
NCT01334047	Ovarian cancer	Cell therapy based on dendritic cells transfected with mRNA	Survivin	Not applicable	Intradermal	Terminated	Oslo University Hospital
NCT00243529	Melanoma	Autologous dendritic cell vaccine	gp100 and tyrosinase	Not applicable	Not applicable	Completed	Radboud University, OIs
NCT00961844	Metastatic Malignant Melanoma	Dendritic cells transfected with hTERT-, survivin- and tumour cell-derived mRNA + ex vivo T cell expansion and reinfusion	Not applicable	Not applicable	Not applicable	Terminated	Steinar Aamda
NCT03897881	High-risk resected cutaneous melanoma	mRNA-4157/V940 (novel mRNA-based personalized cancer vaccine)	Individualized mRNA encoding for up to 34 neoantigens	mRNA-4157 (V940) + pembrolizumab (Keytruda)	Intramuscular	Improved recurrence-free survival with combination vs. pembrolizumab alone. Currently recruiting, estimated completion: September 9, 2029.	ModernaTX, Inc. Collaborator: Merck Sharp & Dohme (subsidiary of Merck & Co.)
NCT03480152	Metastatic gastrointestinal cancers (esophageal squamous carcinoma, gastric adenocarcinoma, pancreatic adenocarcinoma, colorectal adenocarcinoma)	Personalized mRNA cancer vaccine	Defined neoantigens, predicted neoepitopes, mutations in driver genes expressed by the patient's cancer	Monotherapy (no combination)	Intramuscular	Not applicable	National Cancer Institute (NCI), OIs
NCT01066390	Melanoma	TriMix-DC	Not specified	Not specified	Intradermal (i.d.) and Intravenous (i.v.)	Completed Phase 1 study on safety and immunogenicity	Bart Neyns, OIs
NCT00204516	Melanoma	mRNA coding for melanoma-associated antigens	gp100, tyrosinase	Not specified	Not specified	Not specified	University Hospital Tuebingen, OIs
NCT00087373	Metastatic melanoma	Recombinant fowlpox-TRICOM vaccine	Not specified	Not specified	DC-vaccine	Phase 2 study, status not specified	National Cancer Institute (NCI), University of Chicago
NCT00514189	Colon Cancer, Gastrointestinal Cancer	Monocyte-derived dendritic cells	AML mRNA + lysate	Not specified	Not specified	Phase -1	MD Anderson Cancer Center
NCT01278914	Prostate Cancer	mRNA-transfected dendritic cells	Not specified	Not specified	Not specified	I/II	Oslo University Hospital
NCT01446731	Metastatic castration-resistant prostate cancer	DC vaccine	Tumor-specific antigens or tumor-associated self-antigens, encoding PSA, PAP, survivin and hTERT antigens	Not specified	Intramuscular (i.m.)	Completed, encoding PSA, PAP, survivin and hTERT antigens	Inge Marie Svane
NCT02140138	Prostate cancer	CV9104 (mRNA-based multivalent cancer immunotherapy)	–	–	Not specified	Terminated	CureVac AG

## 9 Conclusion

Cancer is declared the deadliest clinicopathological condition encompassing the whole globe owing to its prevalence and impact. However, recent advancements in molecular medicine, particularly the development of mRNA vaccines and cancer immunotherapy, offer great promise for effectively treating cancer. Numerous clinical trials are underway for the formulation of novel vaccines against cancer, either as standalone or administered in combinational therapies. Although, initial vaccine trials have reported promising results, a substantial follow-up alongside critical improvements is still needed before a finalized product can be made accessible to the general public. Hence, enhancing the vaccine technology to boost efficacy and safety remains a pivotal challenge to be addressed shortly.

## References

[1] Soerjomataram I Bray F. Planning for tomorrow: global cancer incidence and the role of prevention 2020–2070. Nat Rev Clin Oncol. (2021) 18:663–72. doi: 10.1038/s41571-021-00514-z 34079102

[2] Gupta S Shukla S. Limitations of immunotherapy in cancer. Cureus. (2022) 14(10):e30856. doi: 10.7759/cureus.30856 36465776 PMC9708058

[3] Wang Y Zhang Z Luo J Han X Wei Y Wei X. mRNA vaccine: a potential therapeutic strategy. Mol cancer. (2021) 20:33. doi: 10.1186/s12943-021-01311-z 33593376 PMC7884263

[4] Heine A Juranek S Brossart P. Clinical and immunological effects of mRNA vaccines in Malignant diseases. Mol cancer. (2021) 20:1–20. doi: 10.1186/s12943-021-01339-1 33722265 PMC7957288

[5] Qu Y Xu J Zhang T Chen Q Sun T Jiang C. Advanced nano-based strategies for mRNA tumor vaccine. Acta Pharm Sin B. (2024) 14:170–89. doi: 10.1016/j.apsb.2023.07.025 PMC10792970 38239240

[6] Wei J Hui AM. The paradigm shift in treatment from Covid-19 to oncology with mRNA vaccines. Cancer Treat Rev. (2022) 107:102405. doi: 10.1016/j.ctrv.2022.102405 35576777 PMC9068246

[7] Al-Haideri M Tondok SB Safa SH Maleki AH Rostami S Jalil AT. CAR-T cell combination therapy: the next revolution in cancer treatment. Cancer Cell Int. (2022) 22:365. doi: 10.1186/s12935-022-02778-6 36419058 PMC9685957

[8] Wu J Wu W Zhou B Li B. Chimeric antigen receptor therapy meets mRNA technology. Trends Biotechnol. (2024) 42:228–40. doi: 10.1016/j.tibtech.2023.08.005 37741706

[9] Wu Q Bazzini AA. Translation and mRNA Stability Control. Annu Rev Biochem. (2023) 92:227–45. doi: 10.1146/annurev-biochem-052621-091808 37001134

[10] Pardi N Hogan MJ Weissman D. Recent advances in mRNA vaccine technology. Curr Opin Immunol. (2020) 65:14–20. doi: 10.1016/j.coi.2020.01.008 32244193

[11] Gao M Zhang Q Feng XH Liu J. Synthetic modified messenger RNA for therapeutic applications. Acta biomaterialia. (2021) 131:1–5. doi: 10.1016/j.actbio.2021.06.020 34133982 PMC8198544

[12] Wadhwa A Aljabbari A Lokras A Foged C Thakur A. Opportunities and challenges in the delivery of mRNA-based vaccines. Pharmaceutics. (2020) 12(2):102. 32013049 10.3390/pharmaceutics12020102 PMC7076378

[13] Zong Y Lin Y Wei T Cheng Q. Lipid nanoparticle (LNP) enables mRNA delivery for cancer therapy. Adv Mater. (2023) 35:2303261. doi: 10.1002/adma.202303261 37196221

[14] Van Hoecke L Roose K. How mRNA therapeutics are entering the monoclonal antibody field. J Trans Med. (2019) 17:54. doi: 10.1186/s12967-019-1804-8 PMC6387507 30795778

[15] Linares-Fernández S Lacroix C Exposito JY Verrier B. Tailoring mRNA vaccine to balance innate/adaptive immune response. Trends Mol Med. (2020) 26:311–23. doi: 10.1016/j.molmed.2019.10.002 31699497

[16] Conry RM LoBuglio AF Wright M Sumerel L Pike MJ Johanning F. Characterization of a messenger RNA polynucleotide vaccine vector. Cancer Res. (1995) 55:1397–400. 7882341

[17] Bukhari MH Syed M Zain S. The differences between traditional vaccines and RNA vaccines: safety, efficacy, reliability and future of COVID-19 vaccines. Lahore, Pakistan: Annals of King Edward Medical University (2021) 27(2). doi: 10.21649/akemu.v27i2.4531

[18] Thess A Grund S Mui BL Hope MJ Baumhof P Fotin-Mleczek M. Sequence-engineered mRNA without chemical nucleoside modifications enables an effective protein therapy in large animals. Mol Ther. (2015) 23:1456–64. doi: 10.1038/mt.2015.103 PMC4817881 26050989

[19] Cafri G Gartner JJ Zaks T Hopson K Levin N Paria BC. mRNA vaccine–induced neoantigen-specific T cell immunity in patients with gastrointestinal cancer. J Clin Invest. (2020) 130:5976–88. doi: 10.1172/JCI134915 PMC7598064 33016924

[20] Esposito M Minnai F Copetti M Miscio G Perna R Piepoli A. Human leukocyte antigen variants associate with BNT162b2 mRNA vaccine response. Commun Med. (2024) 4:63. doi: 10.1038/s43856-024-00490-2 38575714 PMC10995155

[21] Maruggi G Ulmer JB Rappuoli R Yu D. Self-amplifying mRNA-based vaccine technology and its mode of action. Cham: Springer International Publishing (2021) p. 31–70. 10.1007/82_2021_233 33861374

[22] Brader ML Williams SJ Banks JM Hui WH Zhou ZH Jin L. Encapsulation state of messenger RNA inside lipid nanoparticles. Biophys J. (2021) 120:2766–70. doi: 10.1016/j.bpj.2021.03.012 PMC8390897 33773963

[23] Firdessa-Fite R Creusot RJ. Nanoparticles versus dendritic cells as vehicles to deliver mRNA encoding multiple epitopes for immunotherapy. Mol Therapy-Methods Clin Dev. (2020) 16:50–62. doi: 10.1016/j.omtm.2019.10.015 PMC6909218 31871957

[24] Kuhn NA Beissert T Simon P Vallazza B Buck J Davies BP. mRNA as a versatile tool for exogenous protein expression. Curr Gene Ther. (2012) 12:347–61. doi: 10.2174/156652312802762536 22827224

[25] Li Y Wang M Peng X Yang Y Chen Q Liu J. mRNA vaccine in cancer therapy: Current advance and future outlook. Clin Trans Med. (2023) 13:e1384. doi: 10.1002/ctm2.v13.8 PMC10447885 37612832

[26] Kim SC Sekhon SS Shin WR Ahn G Cho BK Ahn JY. Modifications of mRNA vaccine structural elements for improving mRNA stability and translation efficiency. Mol Cell toxicol. (2022) 1:1–8. doi: 10.1007/s13273-021-00171-4 PMC8450916 34567201

[27] Nazli O Bozdag AD Tansug T Kir R Kaymak E. The diagnostic importance of CEA and CA 19-9 for the early diagnosis of pancreatic carcinoma. Hepato-gastroenterology. (2000) 47:1750–2. 11149048

[28] Satake K Chung YS Yokomatsu H Nakata B Tanaka H Sawada T. A clinical evaluation of various tumor markers for the diagnosis of pancreatic cancer. Int J pancreatol. (1990) 7:25–36. doi: 10.1007/BF02924217 2081926

[29] Imaoka H Mizuno N Hara K Hijioka S Tajika M Tanaka T. Prognostic impact of carcinoembryonic antigen (CEA) on patients with metastatic pancreatic cancer: A retrospective cohort study. Pancreatology. (2016) 16(5):859–64. 10.1016/j.pan.2016.05.007 27256641

[30] Omar N Yan B Salto-Tellez M. HER2: An emerging biomarker in non-breast and non-gastric cancers. Pathogenesis. (2015) 2:1–9. doi: 10.1016/j.pathog.2015.05.002

[31] Bryant KL Mancias JD Kimmelman AC Der CJ. KRAS: feeding pancreatic cancer proliferation. Trends Biochem Sci. (2014) 39(2):91–100. doi: 10.1016/j.tibs.2013.12.004 PMC3955735 24388967

[32] Eser S Schnieke A Schneider G Saur D. Oncogenic KRAS signalling in pancreatic cancer. Br J Cancer. (2014) 111(5):817–22. doi: 10.1038/bjc.2014.215 PMC4150259 24755884

[33] Ordóñez NG. Application of mesothelin immunostaining in tumor diagnosis. Am J Surg pathol. (2003) 27:1418–28. doi: 10.1097/00000478-200311000-00003 14576474

[34] Argani P Iacobuzio-Donahue C Ryu B Rosty C Goggins M Wilentz RE. Mesothelin is overexpressed in the vast majority of ductal adenocarcinomas of the pancreas: identification of a new pancreatic cancer marker by serial analysis of gene expression (SAGE). Clin Cancer Res. (2001) 7:3862–8. 11751476

[35] Qu CF Li Y Song YJ Rizvi SM Raja C Zhang D. MUC1 expression in primary and metastatic pancreatic cancer cells for *in vitro* treatment by 213Bi-C595 radioimmunoconjugate. Br J cancer. (2004) 91:2086–93. doi: 10.1038/sj.bjc.6602232 PMC2409789 15599383

[36] Li D Xie K Wolff R Abbruzzese JL. Pancreatic cancer. Lancet. (2004) 363:1049–57. doi: 10.1016/S0140-6736(04)15841-8 15051286

[37] Mello SS Flowers BM Mazur PK Lee JJ Müller F Denny SK. Multifaceted role for p53 in pancreatic cancer suppression. Proc Natl Acad Sci. (2023) 120(10):e2211937120. 36848578 10.1073/pnas.2211937120 PMC10013849

[38] Konduri S Colon J Baker CH Safe S Abbruzzese JL Abudayyeh A. Tolfenamic acid enhances pancreatic cancer cell and tumor response to radiation therapy by inhibiting survivin protein expression. Mol Cancer Ther. (2009) 8:533–42. doi: 10.1158/1535-7163.MCT-08-0405 19258429

[39] Seki K Suda T Aoyagi Y Sugawara S Natsui M Motoyama H. Diagnosis of pancreatic adenocarcinoma by detection of human telomerase reverse transcriptase messenger RNA in pancreatic juice with sample qualification. Clin Cancer Res. (2001) 7:1976–81. 11448913

[40] Doi Y Yashiro M Yamada N Amano R Noda S Hirakawa K. VEGF-A/VEGFR-2 signaling plays an important role for the motility of pancreas cancer cells. Ann Surg Oncol. (2012) 19:2733–43. doi: 10.1245/s10434-011-2181-6 22207048

[41] Costache MI Ioana M Iordache S Ene D Costache CA Săftoiu A. VEGF expression in pancreatic cancer and other malignancies: a review of the literature. Romanian J Internal Med. (2015) 53(3):199–208. 10.1515/rjim-2015-0027 26710495

[42] Davis L Tarduno A Lu YC. Neoantigen-reactive T cells: the driving force behind successful melanoma immunotherapy. Cancers. (2021) 13:6061. doi: 10.3390/cancers13236061 34885172 PMC8657037

[43] Berset M Cerottini JP Guggisberg D Romero P Burri F Rimoldi D. Expression of melan-a/MART-1 antigen as a prognostic factor in primary cutaneous melanoma. Int J cancer. (2001) 95:73–7. doi: 10.1002/1097-0215(20010120)95:1<73::AID-IJC1013>3.0.CO;2-S 11241315

[44] Fernandes BF Odashiro AN Saraiva VS Logan P Antecka E Burnier MN Jr. Immunohistochemical expression of melan-A and tyrosinase in uveal melanoma. J carcinogen. (2007) 6:6. doi: 10.1186/1477-3163-6-6 PMC1864985 17445277

[45] Ohsie SJ Sarantopoulos GP Cochran AJ Binder SW. Immunohistochemical characteristics of melanoma. J cutaneous pathol. (2008) 35:433–44. doi: 10.1111/j.1600-0560.2007.00891.x 18399807

[46] Mann JE Hasson N Su DG Adeniran AJ Smalley KS Djureinovic D. GP100 expression is variable in intensity in melanoma. Cancer Immunology Immunother. (2024) 73(10):191. 10.1007/s00262-024-03776-5 PMC11303354 39105816

[47] Barrow C Browning J MacGregor D Davis ID Sturrock S Jungbluth AA. Tumor antigen expression in melanoma varies according to antigen and stage. Clin Cancer Res. (2006) 12:764–71. doi: 10.1158/1078-0432.CCR-05-1544 16467087

[48] Giavina-Bianchi MH Giavina-Bianchi PF Festa C. Melanoma: tumor microenvironment and new treatments. Anais brasileiros dermatol. (2017) 92:156–66. doi: 10.1590/abd1806-4841.20176183 PMC5429098 28538872

[49] Ruault M van der Bruggen P Brun ME Boyle S Roizès G Sario AD. New BAGE (B melanoma antigen) genes mapping to the juxtacentromeric regions of human chromosomes 13 and 21 have a cancer/testis expression profile. Eur J Hum Genet. (2002) 10:833–40. doi: 10.1038/sj.ejhg.5200891 12461691

[50] Hugdahl E Kalvenes MB Mannelqvist M Ladstein RG Akslen LA. Prognostic impact and concordance of TERT promoter mutation and protein expression in matched primary and metastatic cutaneous melanoma. Br J cancer. (2018) 118:98–105. doi: 10.1038/bjc.2017.384 29123258 PMC5765228

[51] Shah S Chen B. Testing for HER2 in breast cancer: a continuing evolution. Pathol Res Int. (2011) 2011:903202. doi: 10.4061/2011/903202 PMC3005907 21188214

[52] Bilous M Ades C Armes J Bishop J Brown R Cooke B. Predicting the HER2 status of breast cancer from basic histopathology data: an analysis of 1500 breast cancers as part of the HER2000 International Study. breast. (2003) 12(2):92–8. 10.1016/s0960-9776(02)00273-4 14659337

[53] Siroy A Abdul-Karim FW Miedler J Fong N Fu P Gilmore H. MUC1 is expressed at high frequency in early-stage basal-like triple-negative breast cancer. Hum pathol. (2013) 44:2159–66. doi: 10.1016/j.humpath.2013.04.010 PMC4167755 23845471

[54] Jansen K Kornfeld L Lennartz M Blessin NC Rico S Kind S. 2355P Carcinoembryonic antigen (CEA) expression in human tumors: A tissue microarray study on 15,413 tumors. Ann Oncol. (2023) 34:S1197. doi: 10.1016/j.annonc.2023.09.1383

[55] Sugita Y Wada H Fujita S Nakata T Sato S Noguchi Y. NY-ESO-1 expression and immunogenicity in Malignant and benign breast tumors. Cancer Res. (2004) 64:2199–204. doi: 10.1158/0008-5472.CAN-03-3070 15026363

[56] Raghavendra A Kalita-de Croft P Vargas AC Smart CE Simpson PT Saunus JM. Expression of MAGE-A and NY-ESO-1 cancer/testis antigens is enriched in triple-negative invasive breast cancers. Histopathology. (2018) 73:68–80. doi: 10.1111/his.2018.73.issue-1 29465777 PMC6635746

[57] Situ D Wang J Ma Y Zhu Z Hu Y Long H. Expression and prognostic relevance of MUC1 in stage IB non-small cell lung cancer. Med Oncol. (2011) 28:596–604. doi: 10.1007/s12032-010-9752-4 21116877

[58] Bag AK Schultz AR Goala P Karreth FA Adeegbe DO. Investigating the role of KLRG1 in regulatory T-cells and implications for anti-tumor immunity in Non-small Cell Lung Cancer. J Immunol. (2021) 206:57–02. doi: 10.4049/jimmunol.206.Supp.57.02

[59] Chen Y Shen L Chen B Han X Yu Y Yuan X. The predictive prognostic values of CBFA2T3, STX3, DENR, EGLN1, FUT4, and PCDH7 in lung cancer. Ann Trans Med. (2021) 9(10):843. doi: 10.21037/atm-21-1392 PMC8184469 34164477

[60] Lynch TJ Bell DW Sordella R Gurubhagavatula S Okimoto RA Brannigan BW. Activating mutations in the epidermal growth factor receptor underlying responsiveness of non–small-cell lung cancer to gefitinib. New Engl J Med. (2004) 350:2129–39. doi: 10.1056/NEJMoa040938 15118073

[61] Al Olayan A Al Hussaini H Jazieh AR. The roles of epidermal growth factor receptor (EGFR) inhibitors in the management of lung cancer. J infect Public Health. (2012) 5:S50–60. doi: 10.1016/j.jiph.2012.09.004 23244189

[62] Reita D Pabst L Pencreach E Guérin E Dano L Rimelen V. Direct targeting KRAS mutation in non-small cell lung cancer: focus on resistance. Cancers. (2022) 14:1321. doi: 10.3390/cancers14051321 35267628 PMC8909472

[63] Baqar AR Wilkins S Staples M Lee CH Oliva K McMurrick P. The role of preoperative CEA in the management of colorectal cancer: A cohort study from two cancer centres. Int J Surge. (2019) 64:10–5. doi: 10.1016/j.ijsu.2019.02.014 30822523

[64] Betge J Schneider NI Harbaum L Pollheimer MJ Lindtner RA Kornprat P. MUC1, MUC2, MUC5AC, and MUC6 in colorectal cancer: expression profiles and clinical significance. Virchows Archiv. (2016) 469:255–65. doi: 10.1007/s00428-016-1970-5 PMC5007278 27298226

[65] Galizia G Lieto E Ferraraccio F De Vita F Castellano P Orditura M. Prognostic significance of epidermal growth factor receptor expression in colon cancer patients undergoing curative surgery. Ann Surg Oncol. (2006) 13:823–35. doi: 10.1245/ASO.2006.05.052 16614884

[66] Dinu D Dobre M Panaitescu E Bîrlă R Iosif C Hoara P. Prognostic significance of KRAS gene mutations in colorectal cancer-preliminary study. J Med Life. (2014) 7:581. 25713627 PMC4316144

[67] Awad H Saleh T Alhesa A Al Shboul S Yousef R Obeid A. Beta Catenin Expression in Colorectal Carcinoma and its relation to survival and prognostic factors. Ann Cancer Res Ther. (2024) 32:10–6. doi: 10.4993/acrt.32.10

[68] Al-Maghrabi H Al-Mansouri Z Al-Maghrabi J. Survivin expression is associated with lymph node metastasis and short survival in patients with colorectal adenocarcinoma. Int J Clin Exp Pathol. (2024) 17:39. doi: 10.62347/ZCUD7995 38455507 PMC10915287

[69] Shia J Klimstra DS Nitzkorski JR Low PS Gonen M Landmann R. Immunohistochemical expression of folate receptor α in colorectal carcinoma: patterns and biological significance. Hum pathol. (2008) 39:498–505. doi: 10.1016/j.humpath.2007.09.013 18342661

[70] Snook AE Magee MS Waldman SA. GUCY2C-targeted cancer immunotherapy: past, present and future. Immunol Res. (2011) 51:161–9. doi: 10.1007/s12026-011-8253-7 PMC6892460 22038530

[71] Yao R Xie C Xia X. Recent progress in mRNA cancer vaccines. Hum Vaccines Immunotherapeutics. (2024) 20(1):2307187. 10.1080/21645515.2024.2307187 PMC10826636 38282471

[72] Zhang L. Multi-epitope vaccines: a promising strategy against tumors and viral infections. Cell Mol Immunol. (2018) 15:182–4. doi: 10.1038/cmi.2017.92 PMC5811687 28890542

[73] Esprit A de Mey W Bahadur Shahi R Thielemans K Franceschini L Breckpot K. Neo-antigen mRNA vaccines. Vaccines. (2020) 8:776. doi: 10.3390/vaccines8040776 33353155 PMC7766040

[74] Loomis KH Lindsay KE Zurla C Bhosle SM Vanover DA Blanchard EL. *In vitro* transcribed mRNA vaccines with programmable stimulation of innate immunity. Bioconjugate Chem. (2018) 29:3072–83. doi: 10.1021/acs.bioconjchem.8b00443 30067354

[75] Abe N Matsumoto K Nishihara M Nakano Y Shibata A Maruyama H. Rolling circle translation of circular RNA in living human cells. Sci Rep. (2015) 5:16435. doi: 10.1038/srep16435 26553571 PMC4639774

[76] Kon E Elia U Peer D. Principles for designing an optimal mRNA lipid nanoparticle vaccine. Curr Opin Biotechnol. (2022) 73:329–36. doi: 10.1016/j.copbio.2021.09.016 PMC8547895 34715546

[77] Li B Luo X Dong Y. Effects of chemically modified messenger RNA on protein expression. Bioconjugate Chem. (2016) 27:849–53. doi: 10.1021/acs.bioconjchem.6b00090 26906521

[78] Schwartz S Bernstein DA Mumbach MR Jovanovic M Herbst RH León-Ricardo BX. Transcriptome-wide mapping reveals widespread dynamic-regulated pseudouridylation of ncRNA and mRNA. Cell. (2014) 159:148–62. doi: 10.1016/j.cell.2014.08.028 PMC4180118 25219674

[79] Uddin MB Wang Z Yang C. Dysregulations of functional RNA modifications in cancer, cancer stemness and cancer therapeutics. Theranostics. (2020) 10:3164. doi: 10.7150/thno.41687 32194861 PMC7053189

[80] Kierzek E Malgowska M Lisowiec J Turner DH Gdaniec Z Kierzek R. The contribution of pseudouridine to stabilities and structure of RNAs. Nucleic Acids Res. (2014) 42:3492–501. doi: 10.1093/nar/gkt1330 PMC3950712 24369424

[81] Karikó K Muramatsu H Welsh FA Ludwig J Kato H Akira S. Incorporation of pseudouridine into mRNA yields superior nonimmunogenic vector with increased translational capacity and biological stability. Mol Ther. (2008) 16:1833–40. doi: 10.1038/mt.2008.200 PMC2775451 18797453

[82] Anderson BR Muramatsu H Jha BK Silverman RH Weissman D Karikó K. Nucleoside modifications in RNA limit activation of 2′-5′-oligoadenylate synthetase and increase resistance to cleavage by RNase L. Nucleic Acids Res. (2011) 39:9329–38. doi: 10.1093/nar/gkr586 PMC3241635 21813458

[83] Anderson BR Muramatsu H Nallagatla SR Bevilacqua PC Sansing LH Weissman D. Incorporation of pseudouridine into mRNA enhances translation by diminishing PKR activation. Nucleic Acids Res. (2010) 38:5884–92. doi: 10.1093/nar/gkq347 PMC2943593 20457754

[84] Nallagatla SR Bevilacqua PC. Nucleoside modifications modulate activation of the protein kinase PKR in an RNA structure-specific manner. Rna. (2008) 14:1201–13. doi: 10.1261/rna.1007408 PMC2390794 18426922

[85] Svitkin YV Cheng YM Chakraborty T Presnyak V John M Sonenberg N. N1-methyl-pseudouridine in mRNA enhances translation through eIF2α-dependent and independent mechanisms by increasing ribosome density. Nucleic Acids Res. (2017) 45:6023–36. doi: 10.1093/nar/gkx135 PMC5449617 28334758

[86] Parr CJ Wada S Kotake K Kameda S Matsuura S Sakashita S. N 1-Methylpseudouridine substitution enhances the performance of synthetic mRNA switches in cells. Nucleic Acids Res. (2020) 48:e35. doi: 10.1093/nar/gkaa070 32090264 PMC7102939

[87] Karikó K Muramatsu H Keller JM Weissman D. Increased erythropoiesis in mice injected with submicrogram quantities of pseudouridine-containing mRNA encoding erythropoietin. Mol Ther. (2012) 20:948–53. doi: 10.1038/mt.2012.7 PMC3345990 22334017

[88] Boros G Miko E Muramatsu H Weissman D Emri E Rózsa D. Transfection of pseudouridine-modified mRNA encoding CPD-photolyase leads to repair of DNA damage in human keratinocytes: A new approach with future therapeutic potential. J Photochem Photobiol B: Biol. (2013) 129:93–9. doi: 10.1016/j.jphotobiol.2013.09.010 PMC3888937 24211294

[89] Karikó K Buckstein M Ni H Weissman D. Suppression of RNA recognition by Toll-like receptors: the impact of nucleoside modification and the evolutionary origin of RNA. Immunity. (2005) 23:165–75. doi: 10.1016/j.immuni.2005.06.008 16111635

[90] Andries O Mc Cafferty S De Smedt SC Weiss R Sanders NN Kitada T. N1-methylpseudouridine-incorporated mRNA outperforms pseudouridine-incorporated mRNA by providing enhanced protein expression and reduced immunogenicity in mammalian cell lines and mice. J Controlled Release. (2015) 217:337–44. doi: 10.1016/j.jconrel.2015.08.051 26342664

[91] Nance KD Meier JL. Modifications in an emergency: the role of N1-methylpseudouridine in COVID-19 vaccines. ACS Cent sci. (2021) 7:748–56. doi: 10.1021/acscentsci.1c00197 PMC8043204 34075344

[92] Freund I Eigenbrod T Helm M Dalpke AH. RNA modifications modulate activation of innate toll-like receptors. Genes. (2019) 10:92. doi: 10.3390/genes10020092 30699960 PMC6410116

[93] Mauger DM Cabral BJ Presnyak V Su SV Reid DW Goodman B. mRNA structure regulates protein expression through changes in functional half-life. Proc Natl Acad Sci. (2019) 116:24075–83. doi: 10.1073/pnas.1908052116 PMC6883848 31712433

[94] Gao Y Fang J. RNA 5-methylcytosine modification and its emerging role as an epitranscriptomic mark. RNA Biol. (2021) 18:117–27. doi: 10.1080/15476286.2021.1950993 PMC8677007 34288807

[95] Song H Zhang J Liu B Xu J Cai B Yang H. Biological roles of RNA m5C modification and its implications in Cancer immunotherapy. biomark Res. (2022) 10:15. doi: 10.1186/s40364-022-00362-8 35365216 PMC8973801

[96] Weng Y Li C Yang T Hu B Zhang M Guo S. The challenge and prospect of mRNA therapeutics landscape. Biotechnol adv. (2020) 40:107534. doi: 10.1016/j.biotechadv.2020.107534 32088327

[97] Xue C Zhao Y Li L. Advances in RNA cytosine-5 methylation: detection, regulatory mechanisms, biological functions and links to cancer. biomark Res. (2020) 8:1–3. doi: 10.1186/s40364-020-00225-0 32944246 PMC7490858

[98] Kormann MS Hasenpusch G Aneja MK Nica G Flemmer AW Herber-Jonat S. Expression of therapeutic proteins after delivery of chemically modified mRNA in mice. Nat Biotechnol. (2011) 29:154–7. doi: 10.1038/nbt.1733 21217696

[99] Zhang T Zhao F Li J Sun X Zhang X Wang H. Programmable RNA 5-methylcytosine (m5C) modification of cellular RNAs by dCasRx conjugated methyltransferase and demethylase. Nucleic Acids Res. (2024) 52:2776–91. doi: 10.1093/nar/gkae110 PMC11014266 38366553

[100] Desrosiers R Friderici K Rottman F. Identification of methylated nucleosides in messenger RNA from Novikoff hepatoma cells. Proc Natl Acad Sci. (1974) 71:3971–5. doi: 10.1073/pnas.71.10.3971 PMC434308 4372599

[101] Wang S Lv W Li T Zhang S Wang H Li X. Dynamic regulation and functions of mRNA m6A modification. Cancer Cell Int. (2022) 22:48. doi: 10.1186/s12935-022-02452-x 35093087 PMC8800407

[102] Lin S Choe J Du P Triboulet R Gregory RI. The m6A methyltransferase METTL3 promotes translation in human cancer cells. Mol Cell. (2016) 62:335–45. doi: 10.1016/j.molcel.2016.03.021 PMC4860043 27117702

[103] Roy B. Effects of mRNA modifications on translation: an overview. RNA Modif: Methods Protoc. (2021) 2298:327–56. doi: 10.1007/978-1-0716-1374-0_20 34085254

[104] Karikó K Muramatsu H Ludwig J Weissman D. Generating the optimal mRNA for therapy: HPLC purification eliminates immune activation and improves translation of nucleoside-modified, protein-encoding mRNA. Nucleic Acids Res. (2011) 39:e142. doi: 10.1093/nar/gkr695 21890902 PMC3241667

[105] Liu A Wang X. The pivotal role of chemical modifications in mRNA therapeutics. Front Cell Dev Biol. (2022) 10:901510. doi: 10.3389/fcell.2022.901510 35912117 PMC9326091

[106] Melamed JR Hajj KA Chaudhary N Strelkova D Arral ML Pardi N. Lipid nanoparticle chemistry determines how nucleoside base modifications alter mRNA delivery. J Controlled Release. (2022) 341:206–14. doi: 10.1016/j.jconrel.2021.11.022 PMC8905090 34801660

[107] Phan T Fan D Melstrom LG. Developing Vaccines in Pancreatic Adenocarcinoma: Trials and Tribulations. Current Oncology. (2024) 12:5746–63. 10.3390/curroncol31090361 PMC11430674 39329989

[108] Xu S Yang K Li R Zhang L. mRNA vaccine era—mechanisms, drug platform and clinical prospection. Int J Mol Sci. (2020) 21:6582. doi: 10.3390/ijms21186582 32916818 PMC7554980

[109] Shuman S. The mRNA capping apparatus as drug target and guide to eukaryotic phylogeny. In: Cold Spring Harbor symposia on quantitative biology, vol. 66. Cold Spring Harbor, New York, USA: Cold Spring Harbor Laboratory Press (2001). 66:301–12. 10.1101/sqb.2001.66.301 12762032

[110] Gu M Lima CD. Processing the message: structural insights into capping and decapping mRNA. Curr Opin Struct Biol. (2005) 15:99–106. doi: 10.1016/j.sbi.2005.01.009 15718140

[111] Lewis JD Izaurflde E. The role of the cap structure in RNA processing and nuclear export. Eur J Biochem. (1997) 247:461–9. doi: 10.1111/j.1432-1033.1997.00461.x 9266685

[112] Furuichi Y Shatkin AJ. Viral and cellular mRNA capping: past and prospects. Advances in virus research. (2000) 55:135–84. doi: 10.1016/S0065-3527(00)55003-9 PMC7131690 11050942

[113] Sonenberg N Hinnebusch AG. Regulation of translation initiation in eukaryotes: mechanisms and biological targets. Cell. (2009) 136:731–45. doi: 10.1016/j.cell.2009.01.042 PMC3610329 19239892

[114] Shuman S. What messenger RNA capping tells us about eukaryotic evolution. Nat Rev Mol Cell Biol. (2002) 3:619–25. doi: 10.1038/nrm880 12154373

[115] Wang SP Deng L Ho CK Shuman S. Phylogeny of mRNA capping enzymes. Proc Natl Acad Sci. (1997) 94:9573–8. doi: 10.1073/pnas.94.18.9573 PMC23221 9275164

[116] Shatkin AJ. Capping of eucaryotic mRNAs. Cell. (1976) 9:645–53. doi: 10.1016/0092-8674(76)90128-8 1017010

[117] Shatkin AJ Manley JL. The ends of the affair: capping and polyadenylation. Nat Struct Biol. (2000) 7:838–42. doi: 10.1038/79583 11017188

[118] Daffis S Szretter KJ Schriewer J Li J Youn S Errett J. 2′-O methylation of the viral mRNA cap evades host restriction by IFIT family members. Nature. (2010) 468:452–6. doi: 10.1038/nature09489 PMC3058805 21085181

[119] Park JE Yi H Kim Y Chang H Kim VN. Regulation of poly (A) tail and translation during the somatic cell cycle. Mol Cell. (2016) 62:462–71. doi: 10.1016/j.molcel.2016.04.007 27153541

[120] Jalkanen AL Coleman SJ Wilusz J. Determinants and implications of mRNA poly (A) tail size–does this protein make my tail look big? In: Seminars in cell & developmental biology, vol. 34. Cambridge, Massachusetts, USA: Academic Press (2014). 36:24–32. doi: 10.1016/j.semcdb.2014.05.018 24910447 PMC4163081

[121] Kieleczawa J. Fundamentals of sequencing of difficult templates—an overview. J biomol tech: JBT. (2006) 17:207. 16870712 PMC2291785

[122] Diebold SS Kaisho T Hemmi H Akira S Reis e Sousa C. Innate antiviral responses by means of TLR7-mediated recognition of single-stranded RNA. Science. (2004) 303:1529–31. doi: 10.1126/science.1093616 14976261

[123] Tanguay RL Gallie DR. Translational efficiency is regulated by the length of the 3′ untranslated region. Mol Cell Biol. (1996) 16:146–56. doi: 10.1128/MCB.16.1.146 PMC230988 8524291

[124] Leppek K Das R Barna M. Functional 5′ UTR mRNA structures in eukaryotic translation regulation and how to find them. Nat Rev Mol Cell Biol. (2018) 19:158–74. doi: 10.1038/nrm.2017.103 PMC5820134 29165424

[125] Vinhas V Freire M Bacellar O Cunha S Rocha H Carvalho EM. Characterization of T cell responses to purified leishmania antigens in subjects infected with Leishmania chagasi. Braz J Med Biol Research= Rev Bras Pesquisas Medicas e Biol. (1994) 27:1199–205. 8000341

[126] Troye-Blomberg M Olerup O Perlmann H Larsson A Elghazali G Fogdell A. Characterization of regulatory T-cell responses in humans induced by the P. Falciparum blood stage antigen Pf155/RESA. Behring Inst Mitt. (1994) 95:97–105. 7755513

[127] Yu J Russell JE. Structural and functional analysis of an mRNP complex that mediates the high stability of human beta-globin mRNA. Mol Cell Biol. (2001) 21(17):5879–5888. doi: 10.1128/MCB.21.17.5879-5888.2001 PMC87307 11486027

[128] Wang Z Kiledjian M. The poly (A)-binding protein and an mRNA stability protein jointly regulate an endoribonuclease activity. Mol Cell Biol. (2000) 20(17):6334–41. doi: 10.1128/.20.17.6334-6341.2000 PMC86108 10938110

[129] Zhang J Liu Y Li C Xiao Q Zhang D Chen Y. Recent advances and innovations in the preparation and purification of *in Vitro-*Transcribed-mRNA-Based molecules. Pharmaceutics. (2023) 15:2182. doi: 10.3390/pharmaceutics15092182 37765153 PMC10536309

[130] Beckert B Masquida B. Synthesis of RNA by *in vitro* transcription. RNA: methods and protocols. Methods Mol Biol (Clifton, NJ). (2011) 703:29–41. doi: 10.1007/978-1-59745-248-9_3 21125481

[131] Thiel V Herold J Schelle B Siddell SG. Infectious RNA transcribed *in vitro* from a cDNA copy of the human coronavirus genome cloned in vaccinia virus. J Gen Virol. (2001) 82:1273–81. doi: 10.1099/0022-1317-82-6-1273 11369870

[132] Pascolo S. Messenger RNA-based vaccines. Expert Opin Biol Ther. (2004) 4:1285–94. doi: 10.1517/14712598.4.8.1285 15268662

[133] Iavarone C O’hagan DT Yu D Delahaye NF Ulmer JB. Mechanism of action of mRNA-based vaccines. Expert Rev Vaccines. (2017) 16:871–81. doi: 10.1080/14760584.2017.1355245 28701102

[134] Pardi N Hogan MJ Porter FW Weissman D. mRNA vaccines—a new era in vaccinology. Nat Rev Drug discover. (2018) 17:261–79. doi: 10.1038/nrd.2017.243 PMC5906799 29326426

[135] Tenchov R Bird R Curtze AE Zhou Q. Lipid nanoparticles─from liposomes to mRNA vaccine delivery, a landscape of research diversity and advancement. ACS nano. (2021) 15:16982–7015. doi: 10.1021/acsnano.1c04996 34181394

[136] Wei J Hui A. Review of ribosome interactions with SARS-CoV-2 and COVID-19 mRNA vaccine. Life. (2022) 12:57. doi: 10.3390/life12010057 35054450 PMC8780073

[137] Lung P Yang J Li Q. Nanoparticle formulated vaccines: opportunities and challenges. Nanoscale. (2020) 12:5746–63. 10.1039/c9nr08958f 32124894

[138] Mei Y Wang X. RNA modification in mRNA cancer vaccines. Clin Exp Med. (2023) 23:1917–31. doi: 10.1007/s10238-023-01020-5 PMC9928499 36788153

[139] Oberli MA Reichmuth AM Dorkin JR Mitchell MJ Fenton OS Jaklenec A. Lipid nanoparticle assisted mRNA delivery for potent cancer immunotherapy. Nano letters. (2017) 17:1326–35. doi: 10.1021/acs.nanolett.6b03329 PMC5523404 28273716

[140] Lee Y Choe J Park OH Kim YK. Molecular mechanisms driving mRNA degradation by m6A modification. Trends Genet. (2020) 36:177–88. doi: 10.1016/j.tig.2019.12.007 31964509

[141] Glover DJ Lipps HJ Jans DA. Towards safe, non-viral therapeutic gene expression in humans. Nat Rev Genet. (2005) 6:299–310. doi: 10.1038/nrg1577 15761468

[142] Qin M Du G Sun X. Recent advances in the noninvasive delivery of mRNA. Accounts Chem Res. (2021) 54:4262–71. doi: 10.1021/acs.accounts.1c00493 34756014

[143] Butt MH Zaman M Ahmad A Khan R Mallhi TH Hasan MM. Appraisal for the potential of viral and nonviral vectors in gene therapy: A review. Genes. (2022) 13:1370. doi: 10.3390/genes13081370 36011281 PMC9407213

[144] Sharma D Arora S Singh J Layek B. A review of the tortuous path of nonviral gene delivery and recent progress. Int J Biol macromol. (2021) 183:2055–73. doi: 10.1016/j.ijbiomac.2021.05.192 PMC8266766 34087309

[145] Li X Le Y Zhang Z Nian X Liu B Yang X. Viral vector-based gene therapy. Int J Mol Sci. (2023) 24:7736. doi: 10.3390/ijms24097736 37175441 PMC10177981

[146] Travieso T Li J Mahesh S Mello JD Blasi M. The use of viral vectors in vaccine development. NPJ Vaccines. (2022) 7:75. doi: 10.1038/s41541-022-00503-y 35787629 PMC9253346

[147] Wang Y Bruggeman KF Franks S Gautam V Hodgetts SI Harvey AR. Is viral vector gene delivery more effective using biomaterials? Adv Healthc Mater. (2021) 10:2001238. doi: 10.1002/adhm.202001238 33191667

[148] Kowalski PS Rudra A Miao L Anderson DG. Delivering the messenger: advances in technologies for therapeutic mRNA delivery. Mol Ther. (2019) 27:710–28. doi: 10.1016/j.ymthe.2019.02.012 PMC6453548 30846391

[149] Yang W Mixich L Boonstra E Cabral H. Polymer-based mRNA delivery strategies for advanced therapies. Adv Healthc Mater. (2023) 12:2202688. doi: 10.1002/adhm.202202688 36785927 PMC11469255

[150] Barenholz YC. Doxil^®^—The first FDA-approved nano-drug: Lessons learned. J Controlled release. (2012) 160:117–34. doi: 10.1016/j.jconrel.2012.03.020 22484195

[151] Sun D Lu ZR. Structure and function of cationic and ionizable lipids for nucleic acid delivery. Pharm Res. (2023) 40:27–46. doi: 10.1007/s11095-022-03460-2 36600047 PMC9812548

[152] Liu X Huang P Yang R Deng H. mRNA Cancer vaccines: construction and boosting strategies. ACS nano. (2023) 17:19550–80. doi: 10.1021/acsnano.3c05635 37819640

[153] Karlsson J Rhodes KR Green JJ Tzeng SY. Poly (beta-amino ester) s as gene delivery vehicles: challenges and opportunities. Expert Opin Drug delivery. (2020) 17:1395–410. doi: 10.1080/17425247.2020.1796628 PMC7658038 32700581

[154] Li M Li Y Peng K Wang Y Gong T Zhang Z. Engineering intranasal mRNA vaccines to enhance lymph node trafficking and immune responses. Acta biomaterialia. (2017) 64:237–48. doi: 10.1016/j.actbio.2017.10.019 29030308

[155] McKinlay CJ Vargas JR Blake TR Hardy JW Kanada M Contag CH. Charge-altering releasable transporters (CARTs) for the delivery and release of mRNA in living animals. Proc Natl Acad Sci. (2017) 114:E448–56. doi: 10.1073/pnas.1614193114 PMC5278438 28069945

[156] Haabeth OA Lohmeyer JJ Sallets A Blake TR Sagiv-Barfi I Czerwinski DK. An mRNA SARS-CoV-2 vaccine employing charge-altering releasable transporters with a TLR-9 agonist induces neutralizing antibodies and T cell memory. ACS Cent Sci. (2021) 7:1191–204. doi: 10.1021/acscentsci.1c00361 PMC8265720 34341771

[157] Wang Y Zhang L Xu Z Miao L Huang L. mRNA vaccine with antigen-specific checkpoint blockade induces an enhanced immune response against established melanoma. Mol Ther. (2018) 26:420–34. doi: 10.1016/j.ymthe.2017.11.009 PMC5835019 29249397

[158] Xu Z Fisher DE. mRNA melanoma vaccine revolution spurred by the COVID-19 pandemic. Front Immunol. (2023) 14:1155728. doi: 10.3389/fimmu.2023.1155728 37063845 PMC10101324

[159] Sharifnia Z Bandehpour M Hamishehkar H Mosaffa N Kazemi B Zarghami N. *In-vitro* transcribed mRNA delivery using PLGA/PEI nanoparticles into human monocyte-derived dendritic cells. Iranian J Pharm Res: IJPR. (2019) 18:1659. doi: 10.22037/ijpr.2019.1100872 PMC7059071 32184837

[160] Patel S Ashwanikumar N Robinson E DuRoss A Sun C Murphy-Benenato KE. Boosting intracellular delivery of lipid nanoparticle-encapsulated mRNA. Nano letters. (2017) 17:5711–8. doi: 10.1021/acs.nanolett.7b02664 PMC5623340 28836442

[161] Xiao Y Shi J. Lipids and the emerging RNA medicines. Chem Rev. (2021) 121:12109–11. doi: 10.1021/acs.chemrev.1c00778 34702039

[162] Schoenmaker L Witzigmann D Kulkarni JA Verbeke R Kersten G Jiskoot W. mRNA-lipid nanoparticle COVID-19 vaccines: Structure and stability. Int J pharm. (2021) 601:120586. doi: 10.1016/j.ijpharm.2021.120586 33839230 PMC8032477

[163] Kulkarni JA Cullis PR van der Meel R. Lipid nanoparticles enabling gene therapies: from concepts to clinical utility. Nucleic Acid Ther. (2018) 28:146–57. doi: 10.1089/nat.2018.0721 29683383

[164] Kulkarni JA Witzigmann D Chen S Cullis PR van der Meel R. Lipid nanoparticle technology for clinical translation of siRNA therapeutics. Accounts Chem Res. (2019) 52:2435–44. doi: 10.1021/acs.accounts.9b00368 31397996

[165] Tilstra G Couture-Senécal J Lau YM Manning AM Wong DS Janaeska WW. Iterative design of ionizable lipids for intramuscular mRNA delivery. J Am Chem Society. (2023) 145:2294–304. doi: 10.1021/jacs.2c10670 36652629

[166] Kim M Jeong M Hur S Cho Y Park J Jung H. Engineered ionizable lipid nanoparticles for targeted delivery of RNA therapeutics into different types of cells in the liver. Sci Adv. (2021) 7:eabf4398. doi: 10.1126/sciadv.abf4398 33637537 PMC7909888

[167] Tang X Zhang Y Han X. Ionizable lipid nanoparticles for mRNA delivery. Adv NanoBiomed Res. (2023) 3:2300006. doi: 10.1002/anbr.202300006

[168] Semple SC Akinc A Chen J Sandhu AP Mui BL Cho CK. Rational design of cationic lipids for siRNA delivery. Nat Biotechnol. (2010) 28:172–6. doi: 10.1038/nbt.1602 20081866

[169] Jayaraman M Ansell SM Mui BL Tam YK Chen J Du X. Maximizing the potency of siRNA lipid nanoparticles for hepatic gene silencing *in vivo* . Angewandte Chemie. (2012) 124:8657–61. doi: 10.1002/ange.201203263 PMC3470698 22782619

[170] Dong Y Love KT Dorkin JR Sirirungruang S Zhang Y Chen D. Lipopeptide nanoparticles for potent and selective siRNA delivery in rodents and nonhuman primates. Proc Natl Acad Sci. (2014) 111:3955–60. doi: 10.1073/pnas.1322937111 PMC3964096 24516150

[171] Maier MA Jayaraman M Matsuda S Liu J Barros S Querbes W. Biodegradable lipids enabling rapidly eliminated lipid nanoparticles for systemic delivery of RNAi therapeutics. Mol Ther. (2013) 21:1570–8. doi: 10.1038/mt.2013.124 PMC3734658 23799535

[172] Hafez IM Maurer N Cullis PR. On the mechanism whereby cationic lipids promote intracellular delivery of polynucleic acids. Gene Ther. (2001) 8:1188–96. doi: 10.1038/sj.gt.3301506 11509950

[173] Sabnis S Kumarasinghe ES Salerno T Mihai C Ketova T Senn JJ. A novel amino lipid series for mRNA delivery: improved endosomal escape and sustained pharmacology and safety in non-human primates. Mol Ther. (2018) 26:1509–19. doi: 10.1016/j.ymthe.2018.03.010 PMC5986714 29653760

[174] Hou X Zaks T Langer R Dong Y. Lipid nanoparticles for mRNA delivery. Nat Rev Mater. (2021) 6:1078–94. doi: 10.1038/s41578-021-00358-0 PMC8353930 34394960

[175] Guevara ML Persano F Persano S. Advances in lipid nanoparticles for mRNA-based cancer immunotherapy. Front Chem. (2020) 8:589959. doi: 10.3389/fchem.2020.589959 33195094 PMC7645050

[176] Kauffman KJ Dorkin JR Yang JH Heartlein MW DeRosa F Mir FF. Optimization of lipid nanoparticle formulations for mRNA delivery *in vivo* with fractional factorial and definitive screening designs. Nano letters. (2015) 15:7300–6. doi: 10.1021/acs.nanolett.5b02497 26469188

[177] Fenton OS Kauffman KJ McClellan RL Kaczmarek JC Zeng MD Andresen JL. Customizable lipid nanoparticle materials for the delivery of siRNAs and mRNAs. Angewandte Chemie Int Edition. (2018) 57:13582–6. doi: 10.1002/anie.201809056 PMC7548314 30112821

[178] Eygeris Y Patel S Jozic A Sahay G. Deconvoluting lipid nanoparticle structure for messenger RNA delivery. Nano letters. (2020) 20:4543–9. doi: 10.1021/acs.nanolett.0c01386 32375002

[179] Álvarez-Benedicto E Farbiak L Ramírez MM Wang X Johnson LT Mian O. Optimization of phospholipid chemistry for improved lipid nanoparticle (LNP) delivery of messenger RNA (mRNA). Biomater sci. (2022) 10:549–59. doi: 10.1039/D1BM01454D PMC9113778 34904974

[180] Zhang C Ma Y Zhang J Kuo JC Zhang Z Xie H. Modification of lipid-based nanoparticles: An efficient delivery system for nucleic acid-based immunotherapy. Molecules. (2022) 27:1943. doi: 10.3390/molecules27061943 35335310 PMC8949521

[181] Larson NR Hu G Wei Y Tuesca AD Forrest ML Middaugh CR. pH-dependent phase behavior and stability of cationic lipid–mRNA nanoparticles. J Pharm Sci. (2022) 111:690–8. doi: 10.1016/j.xphs.2021.11.004 34774918

[182] Zhang L More KR Ojha A Jackson CB Quinlan BD Li H. Effect of mRNA-LNP components of two globally-marketed COVID-19 vaccines on efficacy and stability. NPJ Vaccines. (2023) 8:156. doi: 10.1038/s41541-023-00751-6 37821446 PMC10567765

[183] Jung HN Lee SY Lee S Youn H Im HJ. Lipid nanoparticles for delivery of RNA therapeutics: Current status and the role of *in vivo* imaging. Theranostics. (2022) 12:7509. doi: 10.7150/thno.77259 36438494 PMC9691360

[184] Mehta M Bui TA Yang X Aksoy Y Goldys EM Deng W. Lipid-based nanoparticles for drug/gene delivery: An overview of the production techniques and difficulties encountered in their industrial development. ACS Mater Au. (2023) 3:600–19. doi: 10.1021/acsmaterialsau.3c00032 PMC10636777 38089666

[185] Carrasco MJ Alishetty S Alameh MG Said H Wright L Paige M. Ionization and structural properties of mRNA lipid nanoparticles influence expression in intramuscular and intravascular administration. Commun Biol. (2021) 4:956. doi: 10.1038/s42003-021-02441-2 34381159 PMC8358000

[186] Chatterjee S Kon E Sharma P Peer D. Endosomal escape: A bottleneck for LNP-mediated therapeutics. Proc Natl Acad Sci. (2024) 121:e2307800120. doi: 10.1073/pnas.2307800120 38437552 PMC10945858

[187] Albertsen CH Kulkarni JA Witzigmann D Lind M Petersson K Simonsen JB. The role of lipid components in lipid nanoparticles for vaccines and gene therapy. Adv Drug delivery Rev. (2022) 188:114416. doi: 10.1016/j.addr.2022.114416 PMC9250827 35787388

[188] Khalil IA Younis MA Kimura S Harashima H. Lipid nanoparticles for cell-specific *in vivo* targeted delivery of nucleic acids. Biol Pharm Bullet. (2020) 43:584–95. doi: 10.1248/bpb.b19-00743 32238701

[189] Hassett KJ Benenato KE Jacquinet E Lee A Woods A Yuzhakov O. Optimization of lipid nanoparticles for intramuscular administration of mRNA vaccines. Mol Therapy-Nucleic Acids. (2019) 15:1–1. doi: 10.1016/j.omtn.2019.01.013 PMC6383180 30785039

[190] Gyanani V Goswami R. Key design features of lipid nanoparticles and electrostatic charge-based lipid nanoparticle targeting. Pharmaceutics. (2023) 15:1184. doi: 10.3390/pharmaceutics15041184 37111668 PMC10144967

[191] Dilliard SA Siegwart DJ. Passive, active and endogenous organ-targeted lipid and polymer nanoparticles for delivery of genetic drugs. Nat Rev Mater. (2023) 8:282–300. doi: 10.1038/s41578-022-00529-7 36691401 PMC9850348

[192] Meyer RA Neshat SY Green JJ Santos JL Tuesca AD. Targeting strategies for mRNA delivery. Mater Today Adv. (2022) 14:100240. doi: 10.1016/j.mtadv.2022.100240

[193] Li M Huang Y Wu J Li S Mei M Chen H. A PEG-lipid-free COVID-19 mRNA vaccine triggers robust immune responses in mice. Mater Horizons. (2023) 10:466–72. doi: 10.1039/D2MH01260J 36468425

[194] Qu Y Xu J Zhang T Chen Q Sun T Jiang C. Advanced nano-based strategies for mRNA tumor vaccine. Acta Pharmaceutica Sinica B. (2024) 14(1):170–89. doi: 10.1016/j.apsb.2023.07.025 PMC10792970 38239240

[195] Karati D Mukherjee S Prajapati B Bose A Paul S Elossaily GM. A review on lipid-polymer hybrid nanocarriers in cancer. J Drug Delivery Sci Technol. (2024) 29:105827. doi: 10.1016/j.jddst.2024.105827

[196] Mukherjee A Waters AK Kalyan P Achrol AS Kesari S Yenugonda VM. Lipid–polymer hybrid nanoparticles as a next-generation drug delivery platform: state of the art, emerging technologies, and perspectives. Int J nanomed. (2019) 19:1937–52. doi: 10.2147/IJN.S198353 PMC6430183 30936695

[197] Awadeen RH Boughdady MF Zaghloul RA Elsaed WM Abu Hashim II Meshali MM. Formulation of lipid polymer hybrid nanoparticles of the phytochemical Fisetin and its *in vivo* assessment against severe acute pancreatitis. Sci Rep. (2023) 13:19110. doi: 10.1038/s41598-023-46215-8 37925581 PMC10625596

[198] Gajbhiye KR Salve R Narwade M Sheikh A Kesharwani P Gajbhiye V. Lipid polymer hybrid nanoparticles: a custom-tailored next-generation approach for cancer therapeutics. Mol Cancer. (2023) 22:160. doi: 10.1186/s12943-023-01849-0 37784179 PMC10546754

[199] Kliesch L Delandre S Gabelmann A Koch M Schulze K Guzmán CA. Lipid–polymer hybrid nanoparticles for mRNA delivery to dendritic cells: impact of lipid composition on performance in different media. Pharmaceutics. (2022) 14:2675. doi: 10.3390/pharmaceutics14122675 36559170 PMC9782540

[200] Mao S Li S Zhang Y Long L Peng J Cao Y. A highly efficient needle-free-injection delivery system for mRNA-LNP vaccination against SARS-CoV-2. Nano Today. (2023) 48:101730. doi: 10.1016/j.nantod.2022.101730 36570700 PMC9767897

[201] Liu H Chen MZ Payne T Porter CJ Pouton CW Johnston AP. Beyond the endosomal bottleneck: understanding the efficiency of mRNA/LNP delivery. Adv Funct Mater. (2024) 34(39):2404510. doi: 10.1002/adfm.202404510

[202] Kauffman KJ Webber MJ Anderson DG. Materials for non-viral intracellular delivery of messenger RNA therapeutics. J Control Release. (2016) 240:227–34. doi: 10.1016/j.jconrel.2015.12.032 26718856

[203] Behr JP. The proton sponge: a trick to enter cells the viruses did not exploit. Chimia. (1997) 51:34. doi: 10.2533/chimia.1997.34

[204] Wu Q Bazzini AA. Translation and mRNA stability control. Annu Rev Biochem. (2023) 92:227–45. doi: 10.1146/annurev-biochem-052621-091808 37001134

[205] Miao L Li L Huang Y Delcassian D Chahal J Han J. Delivery of mRNA vaccines with heterocyclic lipids increases anti-tumor efficacy by STING-mediated immune cell activation. Nat Biotechnol. (2019) 37:1174–85. doi: 10.1038/s41587-019-0247-3 31570898

[206] He M Huang L Hou X Zhong C Bachir ZA Lan M. Efficient ovalbumin delivery using a novel multifunctional micellar platform for targeted melanoma immunotherapy. Int J Pharma. (2019) 560:1–0. doi: 10.1016/j.ijpharm.2019.01.027 30677484

[207] Neill B Romero AR Fenton OS. Advances in nonviral mRNA delivery materials and their application as vaccines for melanoma therapy. ACS Appl Bio Mater. (2023) 7(8):4894–913. doi: 10.1021/acsabm.3c00721 PMC11220486 37930174

[208] Islam MA Xu Y Tao W Ubellacker JM Lim M Aum D. Author Correction: Restoration of tumour-growth suppression in vivo via systemic nanoparticle-mediated delivery of PTEN mRNA. Nat Biomed Eng. (2018) 2(12):968. doi: 10.1038/s41551-018-0331-x 31015729

[209] Xiao Y Chen J Zhou H Zeng X Ruan Z Pu Z. Combining p53 mRNA nanotherapy with immune checkpoint blockade reprograms the immune microenvironment for effective cancer therapy. Nat Commun. (2022) 13:758. doi: 10.1038/s41467-022-28279-8 35140208 PMC8828745

[210] Echaide M Chocarro de Erauso L Bocanegra A Blanco E Kochan G Escors D. mRNA vaccines against SARS-CoV-2: Advantages and caveats. Int J Mol Sci. (2023) 24:5944. doi: 10.3390/ijms24065944 36983017 PMC10051235

[211] Lorentzen CL Haanen JB Met Ö Svane IM. Clinical advances and ongoing trials of mRNA vaccines for cancer treatment. Lancet Oncol. (2022) 23:e450–8. doi: 10.1016/S1470-2045(22)00372-2 PMC9512276 36174631

[212] Boehm DT Landreth KM Sen-Kilic E Lee KS Misra B Bobbala S. Intratumoral administration of mRNA-1273 vaccine delays melanoma growth in mice. bioRxiv. (2024). doi: 10.1101/2024.05.06.592840 PMC11825918 39948424

[213] Curran MA Montalvo W Yagita H Allison JP. PD-1 and CTLA-4 combination blockade expands infiltrating T cells and reduces regulatory T and myeloid cells within B16 melanoma tumors. Proc Natl Acad Sci. (2010) 107:4275–80. doi: 10.1073/pnas.0915174107 PMC2840093 20160101

[214] Kreiter S Vormehr M Van de Roemer N Diken M Löwer M Diekmann J. Mutant MHC class II epitopes drive therapeutic immune responses to cancer. Nature. (2015) 520:692–6. doi: 10.1038/nature14426 PMC4838069 25901682

[215] Chen J Ye Z Huang C Qiu M Song D Li Y. Lipid nanoparticle-mediated lymph node–targeting delivery of mRNA cancer vaccine elicits robust CD8+ T cell response. Proc Natl Acad Sci. (2022) 119:e2207841119. doi: 10.1073/pnas.2207841119 35969778 PMC9407666

[216] Sharbi-Yunger A Grees M Cafri G Bassan D Eichmüller SB Tzehoval E. A universal anti-cancer vaccine: Chimeric invariant chain potentiates the inhibition of melanoma progression and the improvement of survival. Int J cancer. (2019) 144:909–21. doi: 10.1002/ijc.31795 30106470

[217] Sahin U Oehm P Derhovanessian E Jabulowsky RA Vormehr M Gold M. An RNA vaccine drives immunity in checkpoint-inhibitor-treated melanoma. Nature. (2020) 585:107–12. doi: 10.1038/s41586-020-2537-9 32728218

[218] Kieser RE Khan S Bejar N Kiss DL. The dawning of a new enterprise: RNA therapeutics for the skin. J Dermatol skin sci. (2023) 5:4. doi: 10.29245/2767-5092/2023/1.1168 38435714 PMC10907068

[219] Ugurel S Röhmel J Ascierto PA Flaherty KT Grob JJ Hauschild A. Survival of patients with advanced metastatic melanoma: the impact of novel therapies. Eur J cancer. (2016) 53:125–34. doi: 10.1016/j.ejca.2015.09.013 26707829

[220] Khattak A Weber JS Meniawy T Taylor MH Ansstas G Kim KB. Distant metastasis-free survival results from the randomized, phase 2 mRNA-4157-P201/KEYNOTE-942 trial. J Clin Oncol. (2023) 41(17):LBA9503. doi: 10.1200/JCO.2023.41.17_suppl.LBA9503

[221] Weide B Pascolo S Scheel B Derhovanessian E Pflugfelder A Eigentler TK. Direct injection of protamine-protected mRNA: results of a phase 1/2 vaccination trial in metastatic melanoma patients. J Immunother. (2009) 32:498–507. doi: 10.1097/CJI.0b013e3181a00068 19609242

[222] Heesen L Jabulowsky R Loquai C Utikal J Gebhardt C Hassel J. A first-in-human phase I/II clinical trial assessing novel mRNA-lipoplex nanoparticles encoding shared tumor antigens for potent melanoma immunotherapy. Ann Oncol. (2017) 28:xi14–5. doi: 10.1093/annonc/mdx711.030

[223] Clifton GT Litton JK Arrington K Ponniah S Ibrahim NK Gall V. Results of a phase Ib trial of combination immunotherapy with a CD8+ T cell eliciting vaccine and trastuzumab in breast cancer patients. Ann Surg Oncol. (2017) 24:2161–7. doi: 10.1245/s10434-017-5844-0 PMC5908243 28315060

[224] Niemi JV Sokolov AV Schiöth HB. Neoantigen vaccines; clinical trials, classes, indications, adjuvants and combinatorial treatments. Cancers. (2022) 14:5163. 36291947 10.3390/cancers14205163 PMC9600771

[225] Crosby EJ Acharya CR Haddad AF Rabiola CA Lei G Wei JP. Stimulation of oncogene-specific tumor-infiltrating T cells through combined vaccine and αPD-1 enable sustained antitumor responses against established HER2 breast cancer. Clin Cancer Res. (2020) 26:4670–81. doi: 10.1158/1078-0432.CCR-20-0389 PMC7483405 32732224

[226] Jiang XT Liu Q. mRNA vaccination in breast cancer: current progress and future direction. J Cancer Res Clin Oncol. (2023) 149:9435–50. doi: 10.1007/s00432-023-04805-z PMC10132791 37100972

[227] Lin X Chen H Xie Y Zhou X Wang Y Zhou J. Combination of CTLA-4 blockade with MUC1 mRNA nanovaccine induces enhanced anti-tumor CTL activity by modulating tumor microenvironment of triple negative breast cancer. Trans Oncol. (2022) 15:101298. doi: 10.1016/j.tranon.2021.101298 PMC8652013 34875483

[228] Crosby EJ Gwin W Blackwell K Marcom PK Chang S Maecker HT. Vaccine-induced memory CD8+ T cells provide clinical benefit in HER2 expressing breast cancer: a mouse to human translational study. Clin Cancer Res. (2019) 25:2725–36. doi: 10.1158/1078-0432.CCR-18-3102 PMC6497539 30635338

[229] De Mey W Esprit A Thielemans K Breckpot K Franceschini L. RNA in cancer immunotherapy: unlocking the potential of the immune system. Clin Cancer Res. (2022) 28:3929–39. doi: 10.1158/1078-0432.CCR-21-3304 PMC9475240 35583609

[230] Mackensen A Koenecke C Haanen J Alsdorf W Desuki A Wagner-Drouet E. 958 BNT211: a phase I/II trial to evaluate safety and efficacy of CLDN6 CAR-T cells and vaccine-mediated *in vivo* expansion in patients with CLDN6-positive advanced solid tumors. J ImmunoTher Cancer. (2021) 9:A1008. doi: 10.1136/jitc-2021-SITC2021.958

[231] Haanen JB Mackensen A Koenecke C Alsdorf W Desuki A Wagner-Drouet E. LBA1 BNT211: a phase I/II trial to evaluate safety and efficacy of CLDN6 CAR-T cells and CARVac-mediated *in vivo* expansion in patients with CLDN6+ advanced solid tumors. Ann Oncol. (2021) 32:S1392. doi: 10.1016/j.annonc.2021.10.216

[232] Gwin WR Hobeika A Osada T Hartman Z Cheng Q Broadwater G. Effect of alphavirus vaccine encoding HER2 during concurrent anti-HER2 therapies on induction of oligoclonal T cell and antibody responses against HER2. J Clin Oncol. (2015) 33(15):3081. doi: 10.1200/jco.2015.33.15_suppl.3081

[233] Tchou J Zhao Y Levine BL Zhang PJ Davis MM Melenhorst JJ. Safety and efficacy of intratumoral injections of chimeric antigen receptor (CAR) T cells in metastatic breast cancer. Cancer Immunol Res. (2017) 5:1152–61. doi: 10.1158/2326-6066.CIR-17-0189 PMC5712264 29109077

[234] Schmidt M Vogler I Derhovanessian E Omokoko T Godehardt E Attig S. 88MO T-cell responses induced by an individualized neoantigen specific immune therapy in post (neo) adjuvant patients with triple negative breast cancer. Ann Oncol. (2020) 31:S276. doi: 10.1016/j.annonc.2020.08.209

[235] Rojas LA Sethna Z Soares KC Olcese C Pang N Patterson E. Personalized RNA neoantigen vaccines stimulate T cells in pancreatic cancer. Nature. (2023) 618:144–50. doi: 10.1038/s41586-023-06063-y PMC10171177 37165196

[236] Kang N Zhang S Wang Y. A personalized mRNA vaccine has exhibited potential in the treatment of pancreatic cancer. Holist Integr Oncol. (2023) 2:18. doi: 10.1007/s44178-023-00042-z 37323470 PMC10248956

[237] Hong J Guo G Wu S Lin S Zhou Z Chen S. Altered MUC1 epitope-specific CTLs: A potential target for immunotherapy of pancreatic cancer. J Leukocyte Biol. (2022) 112:1577–90. doi: 10.1002/JLB.5MA0922-749R 36222123

[238] Abou-Alfa GK Chapman PB Feilchenfeldt J Brennan MF Capanu M Gansukh B. Targeting mutated K-ras in pancreatic adenocarcinoma using an adjuvant vaccine. American journal of clinical oncology. (2011) 34:321–5. 10.1097/COC.0b013e3181e84b1f 20686403

[239] Suso EM Dueland S Rasmussen AM Vetrhus T Aamdal S Kvalheim G. hTERT mRNA dendritic cell vaccination: complete response in a pancreatic cancer patient associated with response against several hTERT epitopes. Cancer Immunol Immunother. (2011) 60:809–18. doi: 10.1007/s00262-011-0991-9 PMC3098983 21365467

[240] Cowzer D Zameer M Conroy M Kolch W Duffy AG. Targeting KRAS in pancreatic cancer. J Personal Med. (2022) 12:1870. doi: 10.3390/jpm12111870 PMC9692903 36579598

[241] Nagasaka M Potugari B Nguyen A Sukari A Azmi AS Ou SH. KRAS inhibitors–yes but what next? Direct targeting of KRAS–vaccines, adoptive T cell therapy and beyond. Cancer Treat Rev. (2021) 101:102309. doi: 10.1016/j.ctrv.2021.102309 34715449

[242] O’Reilly EM Wainberg ZA Weekes CD Furqan M Kasi PM Devoe CE. AMPLIFY-201, a first-in-human safety and efficacy trial of adjuvant ELI-002 2P immunotherapy for patients with high-relapse risk with KRAS G12D-or G12R-mutated pancreatic and colorectal cancer. J Clin Oncol. (2023) 41(Suppl 16):2528. doi: 10.1200/JCO.2023.41.16_suppl.2528

[243] Sebastian M Papachristofilou A Weiss C Früh M Cathomas R Hilbe W. Phase Ib study evaluating a self-adjuvanted mRNA cancer vaccine (RNActive^®^) combined with local radiation as consolidation and maintenance treatment for patients with stage IV non-small cell lung cancer. BMC cancer. (2014) 14:1–0. doi: 10.1186/1471-2407-14-748 25288198 PMC4195907

[244] Yang R Yu S Xu T Zhang J Wu S. Emerging role of RNA sensors in tumor microenvironment and immunotherapy. J Hematol Oncol. (2022) 15:43. doi: 10.1186/s13045-022-01261-z 35413927 PMC9006576

[245] Sebastian M Schröder A Scheel B Hong HS Muth A von Boehmer L. A phase I/IIa study of the mRNA-based cancer immunotherapy CV9201 in patients with stage IIIB/IV non-small cell lung cancer. Cancer immunol Immunother. (2019) 68:799–812. doi: 10.1007/s00262-019-02315-x 30770959 PMC11028316

[246] Papachristofilou A Hipp MM Klinkhardt U Früh M Sebastian M Weiss C. Phase Ib evaluation of a self-adjuvanted protamine formulated mRNA-based active cancer immunotherapy, BI1361849 (CV9202), combined with local radiation treatment in patients with stage IV non-small cell lung cancer. J immunother cancer. (2019) 7:1–4. doi: 10.1186/s40425-019-0520-5 30736848 PMC6368815

[247] Morse MA Nair SK Mosca PJ Hobeika AC Clay TM Deng Y. Immunotherapy with autologous, human dendritic cells transfected with carcinoembryonic antigen mRNA. Cancer Invest. (2003) 21:341–9. doi: 10.1081/CNV-120018224 12901279

[248] Kiousi E Lyraraki V Mardiki GL Stachika N Damianou AK Malainou CP. Progress and Challenges of Messenger RNA Vaccines in the Therapeutics of NSCLC. Cancers. (2023) 15(23):5589. 38067293 10.3390/cancers15235589 PMC10705317

[249] Chen R Liu W Brown DM Bong YS He J Shen D. A pan-ras mRNA vaccine elicits specific immune responses and inhibits tumor growth in the mouse model of colon cancer. Cancer Res. (2023) 83:5738. doi: 10.1158/1538-7445.AM2023-5738

[250] Hewitt SL Bailey D Zielinski J Apte A Musenge F Karp R. Intratumoral IL12 mRNA therapy promotes TH1 transformation of the tumor microenvironment. Clin Cancer Res. (2020) 26:6284–98. doi: 10.1158/1078-0432.CCR-20-0472 32817076

[251] Li Y Ma X Yue Y Zhang K Cheng K Feng Q. Rapid surface display of mRNA antigens by bacteria-derived outer membrane vesicles for a personalized tumor vaccine. Adv Mater. (2022) 34:2109984. doi: 10.1002/adma.202109984 35315546

[252] Liu L Chen J Zhang H Ye J Moore C Lu C. Concurrent delivery of immune checkpoint blockade modulates T cell dynamics to enhance neoantigen vaccine-generated antitumor immunity. Nat cancer. (2022) 3(4):437–52. 10.1038/s43018-022-00352-7 PMC9050907 35393580

